# Dynamic Analysis of a Cross-Lingual Coupled Rumor Propagation Model with Response Delay in Online Social Networks

**DOI:** 10.3390/e28060691

**Published:** 2026-06-15

**Authors:** Zhengbin Wang, Xiaoming Wang, Yaguang Lin, Zekun Liu

**Affiliations:** 1 School of Computer, Qinghai Normal University, Xining 810016, China; 2 School of Artificial Intelligence and Computer Science, Shaanxi Normal University, Xi’an 710119, China

**Keywords:** cross-lingual coupled rumor propagation, response delay, online social networks, basic reproduction number, Hopf bifurcation, mean-field model

## Abstract

As online social networks (OSNs) evolve into multilingual ecosystems, rumors can cross language boundaries through translation and bilingual re-expression, increasing governance difficulty. To characterize cross-lingual coupling and response delay, this study proposes a time-delay S2LCHR dynamical model for bilingual OSNs, in which the coupled spreader state *C* describes cross-lingual coupled rumor transmission and a fixed response delay represents cross-lingual comprehension, judgment, and re-expression. The basic reproduction number $R_{0}$ is derived using the next-generation matrix method. Lyapunov analysis, the Routh–Hurwitz criterion, characteristic-equation analysis, and numerical simulations are combined to examine equilibrium stability, delay-induced Hopf bifurcation, parameter sensitivity, and social-impact indicators. A real-world aggregate trend-fitting case study using English–Spanish COVID-19-related tweet data is further conducted to assess empirical plausibility. The results show that $R_{0}$ determines the threshold between rumor extinction and persistence in the delay-free system, while an excessive response delay can destabilize the rumor-prevailing equilibrium and induce bounded oscillatory behavior. Sensitivity and social-impact analyses indicate that α and β promote rumor persistence, whereas σ and φ associated with state *C* are key inhibitory factors. These findings suggest that coupled spreaders should be prioritized in cross-lingual rumor governance.

## 1. Introduction

Rumors spread rapidly and often remain concealed, readily amplifying social panic and disrupting the order of public opinion [1,2,3,4]. As OSNs evolve from monolingual communication to multilingual ecosystems, rumors can also cross language boundaries through translation, paraphrasing, and re-expression, further expanding their reach and increasing the difficulty of governance [5,6]. Compared with the monolingual setting, the complexity of multilingual rumor propagation lies in the coupled propagation process that connects different language transmission pathways [7]. Meanwhile, this coupled propagation process is usually not completed instantaneously, but is accompanied by response delay arising from cognitive processing and behavioral preparation; such delay may further alter the stability and evolutionary trajectory of the system [8,9]. Therefore, to characterize rumor diffusion in bilingual OSNs more realistically, it is necessary to consider both the cross-lingual coupled propagation mechanism and the response-delay effect.

Considerable progress has been made in the modeling and analysis of rumor propagation. First, in the general modeling of rumor dynamics, early studies started from classical models such as the DK [10,11] and MT [12] models, thereby laying the foundation for subsequent research. Thereafter, a large body of work further incorporated mechanisms such as forgetting and hesitation into rumor propagation models, promoting their extension toward multi-state frameworks [13]. For example, Wang et al. proposed an S2IR rumor propagation model in a multilingual environment, in which the population was divided into ignorants, two types of spreaders, and stiflers [1]. In addition, some studies have introduced psychological factors, familiarity, or behavioral mechanisms into the system so as to enhance the explanatory power of the models for real rumor-spreading behavior [14,15]. Jia et al. proposed a rumor propagation dynamical model based on adversarial behavior and evolutionary game theory, in which users’ real-time preferences, the antagonistic relationship between the rumor group and the anti-rumor group, and users’ strategy choices were jointly incorporated into the system, and behavioral states such as wavering, rumor, and anti-rumor were defined to characterize the rumor propagation process more realistically [12].

Second, in studies on multilingual rumor propagation, scholars have not only expanded the propagation states but have also introduced cross-lingual transmission pathways on the basis of monolingual models to characterize information migration and cross-transmission processes in multilingual environments [16]. The research introduced a cross-transmission mechanism into heterogeneous networks [17,18,19]. They not only added new states to the monolingual model but also characterized the multilingual propagation process by introducing additional transmission pathways [20]. Lei et al. pointed out that multilingual rumor data often exhibit discrete features, and therefore proposed a discrete multilingual rumor propagation model and studied it in conjunction with optimal control [5]. These studies indicate that multilingual environments significantly affect the threshold, stability, and governance effectiveness of rumor propagation [21,22,23].

Third, delay, bifurcation, and control issues have also become important directions in rumor dynamics in recent years. Relevant studies have shown that propagation delay, recovery delay, media response delay, and control execution delay can all significantly alter the stability structure of a system and may induce Hopf bifurcation and periodic oscillations [24,25]. For example, Niu et al. constructed a fractional-order delayed IDSR model and analyzed the stability and Hopf bifurcation near the positive equilibrium [26]. Zhang et al. introduced recovery delay and saturated control into a reaction-diffusion rumor propagation model, revealing that the system may lose stability and exhibit periodic oscillations once the delay exceeds a critical value [9]. In addition, some studies introduced delay and optimal control into bilingual heterogeneous networks and discussed the inhibitory effect of intervention intensity on spreader density [8].

Although existing studies have produced substantial results in multilingual rumor propagation and delayed rumor dynamics, several key issues remain insufficiently addressed in bilingual OSNs [27,28]. First, many multilingual rumor models treat different language groups as parallel propagation channels or distinguish spreaders only by language category, but do not explicitly characterize the coupled spreader state that bridges two language communities and amplifies rumor diffusion [29]. Second, existing delay studies mainly focus on general propagation delay, recovery delay, media response delay, or control execution delay, whereas the response delay caused by cross-lingual comprehension, judgment, and re-expression has rarely been assigned to the transition from monolingual exposure to cross-lingual coupled spreading [26,30]. Third, existing threshold and stability analyses usually clarify whether rumors persist or die out, but they seldom further identify which propagation state should be prioritized for intervention in a multilingual rumor-spreading system [31,32].

To address these gaps, this paper proposes a time-delay S2LCHR model for cross-lingual coupled rumor propagation in bilingual OSNs. The model explicitly introduces the coupled spreader state *C*, the coupled hesitant state *H*, and a fixed response delay in the transition from $L_{1}$ and $L_{2}$ to *C*. It further links threshold analysis, delay-induced Hopf bifurcation, parameter sensitivity, social-impact indicators, and real-world aggregate trend-fitting to identify key intervention targets [33,34,35,36,37].

The main contributions of this study are summarized as follows:

1. We develop a time-delay S2LCHR rumor-propagation model for bilingual OSNs by explicitly incorporating the coupled spreader state *C*, the coupled hesitant state *H*, and a fixed response delay associated with cross-lingual comprehension, judgment, and bilingual re-expression. This model characterizes the transition from monolingual exposure to cross-lingual coupled spreading and captures the bridging role of bilingual spreaders between two language communities.
2. We derive the basic reproduction number $R_{0}$ by using the next-generation matrix method, analyze the stability of the rumor-free and rumor-prevailing equilibria, and investigate the effect of response delay on the local stability of the rumor-prevailing equilibrium. In particular, we show that an excessive response delay may induce stability switching, local Hopf bifurcation, and bounded oscillatory behavior in cross-lingual rumor propagation.
3. By combining $R_{0}$-based sensitivity analysis, social-impact indicators, and a real-world aggregate trend-fitting case study, we identify the parameters associated with the coupled spreader state as key intervention targets for reducing outbreak intensity and cumulative damage.

The remainder of this paper is organized as follows. Section 2 presents the formulation of the time-delay S2LCHR model. Section 3 investigates the equilibria, threshold dynamics, stability, and Hopf bifurcation of the model. Section 4 provides numerical simulations, sensitivity analysis, social-impact assessment, and a real-world aggregate trend-fitting case study. Finally, Section 5 concludes the paper.

## 2. Formulation of the Time-Delay S2LCHR Model

This section presents the construction of the model in detail, including the state classification and notation, the basic assumptions, and the state transition mechanisms. On this basis, we further establish the time-delay dynamical system, thereby providing a theoretical foundation for the subsequent stability analysis of the equilibria.

### 2.1. State Classification and Basic Assumptions

First, to characterize the coupled rumor propagation behavior across different language communities in a bilingual environment, we divide user nodes in OSNs into the following six mutually exclusive states [38]:

1. Ignorants *S* denote users who have not yet been exposed to rumor information;
2. The monolingual exposed state $L_{1}$ denotes users who are exposed to the rumor in the first language and remain in an exposed state;
3. The monolingual exposed state $L_{2}$ denotes users who are exposed to the rumor in the second language and remain in an exposed state;
4. The coupled spreader state *C* denotes users who possess cross-lingual ability and choose to spread the rumor in a bilingual form;
5. The coupled hesitant state *H* denotes users who waver between propagation and withdrawal, question the rumor, or remain in a re-decision stage;
6. The forgetting state *R* denotes users who withdraw from the current propagation process due to the forgetting mechanism and other factors.

For ease of description and analysis, let N(t) denote the total number of user nodes in OSNs at time *t*. After normalization, let S(t), $L_{1}\left(t\right)$, $L_{2}\left(t\right)$, C(t), H(t), and R(t) denote the proportions of user nodes in the above six states relative to N(t) at time *t*, respectively. Meanwhile, we make the following assumptions:

1. All user nodes in the current OSNs are in one of the above six basic states. To simplify the analysis, we assume that the total size N(t) of the OSNs remains constant during the period under consideration. Therefore, we set Λ=μ, and obtain $S\left(t\right)+L_{1}\left(t\right)+L_{2}\left(t\right)+C\left(t\right)+H\left(t\right)+R\left(t\right)=1,\forall t\geq 0$;
2. Considering the openness and fluidity of OSNs, namely, the entry of new users and the departure of existing users, we assume that new users enter the network at rate Λ and are all assigned to the ignorant state *S* by default. Meanwhile, users in each state leave the network at rate μ (e.g., because existing users deactivate their accounts or become inactive);
3. Rumor-exposed individuals and rumor spreaders are not identical concepts; the former become the latter only with a certain probability.

### 2.2. State Transition Mechanisms and Parameter Descriptions

Based on the above definitions, the propagation mechanism of the S2LCHR model is described as follows.

1. When an ignorant individual *S* comes into contact with rumor information in different language forms, the individual enters the corresponding monolingual exposed state: when the individual contacts the rumor in the first language form, the individual moves to $L_{1}$ at transition rate α; when the individual contacts the rumor in the second language form, the individual moves to $L_{2}$ at transition rate β. In addition, the rate at which users in the monolingual exposed state $L_{1}$ choose to engage in monolingual rumor spreading is denoted by $\eta_{1}$, and the corresponding rate for users in the monolingual exposed state $L_{2}$ is denoted by $\eta_{2}$. It should be emphasized that this monolingual spreading behavior does not change the individual’s state; $\eta_{1}$ and $\eta_{2}$ are introduced only to characterize the spreading activity of $L_{1}$ and $L_{2}$, respectively, and to affect the incidence terms [39,40];
2. Users in the monolingual exposed state $L_{1}$ move to the coupled spreader state *C* at transition rate $\theta_{1}$ after developing the intention for cross-lingual propagation and completing comprehension, judgment, and re-expression preparation. Similarly, the transition rate from the monolingual exposed state $L_{2}$ to the coupled spreader state *C* is $\theta_{2}$. Considering that cross-lingual comprehension and re-expression involve a non-negligible response delay, we introduce a fixed delay τ into the transition from $L_{1}$ or $L_{2}$ to *C*;
3. Due to factors such as skepticism, fatigue, or external intervention, users in the coupled spreader state move to the coupled hesitant state *H* at transition rate σ, and then make a renewed decision between “continuing propagation” and “withdrawing from propagation”: users in state *H* return to the coupled spreader state *C* at transition rate ψ, and withdraw from propagation and move to the forgetting state *R* at transition rate ζ. In addition, also due to factors such as skepticism, fatigue, or external intervention, users in states $L_{1}$, $L_{2}$, and *C* move directly to the forgetting state *R* at transition rates γ, δ, and φ, respectively;
4. Given the openness of OSNs, new users enter the network (e.g., through new registration) at rate Λ, and are initially in the ignorant state *S*. Meanwhile, users in each state leave the network at a uniform natural removal rate μ (e.g., through account cancellation or inactivity).

**Table 1:** Parameter definitions of the S2LCHR model.

Category	Symbol	Description
Inflow/Outflow Parameters	Λ	Inflow rate of new users into the current network
	μ	Removal rate of existing users from the current network
Contact and Propagation Parameters	α	Transition rate at which an ignorant individual moves to state $L_{1}$ after contacting the rumor in the first language form
	β	Transition rate at which an ignorant individual moves to state $L_{2}$ after contacting the rumor in the second language form
	$\eta_{1}$	Activity coefficient for users in the first-language monolingual exposed state $L_{1}$ who choose to conduct monolingual rumor propagation in the first language
	$\eta_{2}$	Activity coefficient for users in the second-language monolingual exposed state $L_{2}$ who choose to conduct monolingual rumor propagation in the second language
Cross-Lingual Coupling Parameters	$\theta_{1}$	Transition rate from the first-language monolingual exposed state $L_{1}$ to the coupled spreader state *C*
	$\theta_{2}$	Transition rate from the second-language monolingual exposed state $L_{2}$ to the coupled spreader state *C*
	τ	Fixed response delay for cross-lingual comprehension and re-expression preparation
Control and Withdrawal Parameters	σ	Transition rate from the coupled spreader state *C* to the coupled hesitant state *H*
	ψ	Reverse transition rate from the coupled hesitant state *H* to the coupled spreader state *C*
	φ	Transition rate from the coupled spreader state *C* to the forgetting state *R*
	γ	Transition rate from the first-language monolingual exposed state $L_{1}$ to the forgetting state *R*
	δ	Transition rate from the second-language monolingual exposed state $L_{2}$ to the forgetting state *R*
	ζ	Transition rate from the coupled hesitant state *H* to the forgetting state *R*

It should be emphasized that the fixed response delay τ adopted in this study does not imply that all users complete cross-lingual comprehension and re-expression after exactly the same amount of time. Rather, τ is interpreted as an effective average response delay at the population level, representing the dominant time lag from monolingual exposure to cross-lingual coupled spreading. We use a fixed delay as a tractable baseline approximation, because it keeps the threshold, stability, and Hopf bifurcation analyses analytically interpretable; a distributed-delay formulation would require an additional delay kernel that is difficult to identify without individual-level response-time data.

In this baseline model, no additional survival factor is imposed on the delayed transition terms, because direct withdrawal from $L_{1}$ and $L_{2}$ is already represented by γ and δ, while a separate waiting-period loss would introduce extra parameters. If such loss is considered, the delayed terms can be generalized as $\theta_{i}L_{i}\left(t-\tau \right)\to \theta_{i}e^{-\kappa_{i}\tau }L_{i}\left(t-\tau \right)$, i=1,2, which would reduce the effective inflow into *C* and may shift the stability boundary or the Hopf bifurcation threshold. All transition rates are defined per unit model time, and this unit can be mapped to minutes, hours, days, weeks, or months depending on the data resolution and rumor type; for example, breaking-news rumors may correspond to short time units, whereas slowly evolving narratives may require longer aggregate time scales.

**Figure 1:**
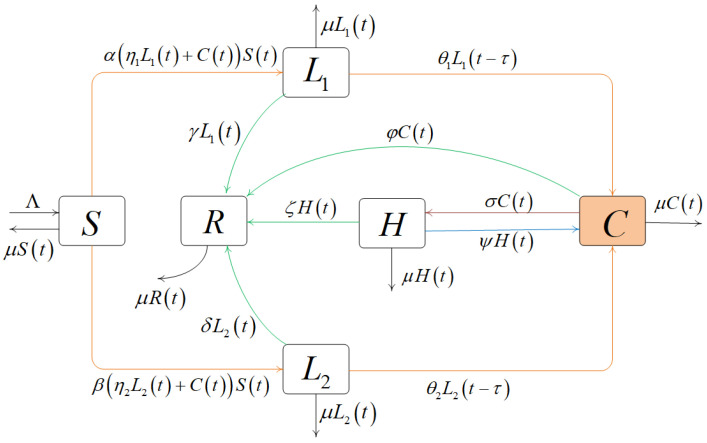
The state transition diagram of the S2LCHR model.

### 2.3. Model Equations

Based on the above state transition mechanisms, we establish the dynamical differential equations of the S2LCHR model, which are given as follows:

(1) $$\left\{\begin{array}{c} \frac{dS(t)}{dt}=\Lambda -\alpha \left[\eta_{1}L_{1}\left(t\right)+C\left(t\right)\right]S\left(t\right)-\beta \left[\eta_{2}L_{2}\left(t\right)+C\left(t\right)\right]S\left(t\right)-\mu S\left(t\right),\\ \frac{dL_{1}\left(t\right)}{dt}=\alpha \left[\eta_{1}L_{1}\left(t\right)+C\left(t\right)\right]S\left(t\right)-\theta_{1}L_{1}\left(t-\tau \right)-\left(\gamma +\mu \right)L_{1}\left(t\right),\\ \frac{dL_{2}\left(t\right)}{dt}=\beta \left[\eta_{2}L_{2}\left(t\right)+C\left(t\right)\right]S\left(t\right)-\theta_{2}L_{2}\left(t-\tau \right)-\left(\delta +\mu \right)L_{2}\left(t\right),\\ \frac{dC(t)}{dt}=\theta_{1}L_{1}\left(t-\tau \right)+\theta_{2}L_{2}\left(t-\tau \right)+\psi H\left(t\right)-\left(\sigma +\varphi +\mu \right)C\left(t\right),\\ \frac{dH(t)}{dt}=\sigma C\left(t\right)-\left(\psi +\zeta +\mu \right)H\left(t\right),\\ \frac{dR(t)}{dt}=\gamma L_{1}\left(t\right)+\delta L_{2}\left(t\right)+\varphi C\left(t\right)+\zeta H\left(t\right)-\mu R\left(t\right), \end{array}\right.$$

For system (1), the initial history functions on t∈[−τ,0] are nonnegative and satisfy the normalization constraint. When τ=0, system (1) reduces to a system of ordinary differential equations.

The S2LCHR system (1) is formulated as a population-level mean-field model, in which S(t), $L_{1}\left(t\right)$, $L_{2}\left(t\right)$, C(t), H(t), and R(t) denote aggregate proportions of users in different propagation states, and interactions are represented by effective average contact and transition rates rather than explicit network edges. This approximation keeps the analysis of cross-lingual coupling, $R_{0}$, equilibrium stability, and Hopf bifurcation analytically interpretable, but it does not explicitly resolve small-world shortcuts, scale-free degree heterogeneity, community structure, or multilayer language topology. In a network-structured extension, $R_{0}$ and the Hopf bifurcation conditions may depend on topological quantities such as the degree distribution, the spectral radius of the adjacency matrix, and the inter-layer coupling strength between language communities.

## 3. Dynamic Behavior Analysis of the S2LCHR Model

In this section, we analyze the equilibria, threshold, stability, and delay-induced bifurcation behavior of system (1) to reveal the long-term dynamical evolution of the bilingual coupled rumor propagation system under different parameter conditions and provide a theoretical basis for the subsequent numerical simulations and discussion of governance strategies.

### 3.1. Equilibrium and Threshold

Setting the right-hand side of system (1) equal to zero and using the normalization condition $S\left(t\right)+L_{1}\left(t\right)+L_{2}\left(t\right)+C\left(t\right)+H\left(t\right)+R\left(t\right)=1$, we obtain the rumor-free equilibrium $E_{0}=\left(1,0,0,0,0,0\right)$ and the rumor-prevailing equilibrium $E_{1}=\left(S^{*},L_{1}^{*},L_{2}^{*},C^{*},H^{*},R^{*}\right)$. For readability, the detailed derivation process of $E_{1}$ can be found in Appendix A.

To ensure that the basic reproduction number $R_{0}$ is mathematically well defined and socially meaningful, the effective coefficient $k_{C}$ must be positive. From $k_{C}=\left(\sigma +\varphi +\mu \right)-\psi k_{H}$, $k_{H}=\frac{\sigma }{\psi +\zeta +\mu }$, we obtain $k_{C}=\frac{(\sigma +\varphi +\mu )(\psi +\zeta +\mu )-\psi \sigma }{\psi +\zeta +\mu }=\frac{\sigma (\zeta +\mu )+(\varphi +\mu )(\psi +\zeta +\mu )}{\psi +\zeta +\mu }$. Therefore, under nonnegative transition rates and ψ+ζ+μ>0, $k_{C}>0$ holds if σ(ζ+μ)+(φ+μ)(ψ+ζ+μ)>0.

In particular, when the natural removal rate μ>0, this condition is automatically satisfied. Behaviorally, this means that the *C*–*H* feedback loop contains effective dissipation through natural departure, direct forgetting from *C*, or withdrawal from *H*. If $k_{C}=0$, the terms $\theta_{1}/k_{C}$ and $\theta_{2}/k_{C}$ in $R_{0}$ become singular; if $k_{C}<0$, the next-generation matrix no longer yields a meaningful positive threshold. Such parameter combinations are therefore excluded from the feasible parameter region.

When τ=0, to compute the basic reproduction number $R_{0}$, we regard the state variables $L_{1}$, $L_{2}$, *C*, and *H* associated with rumor propagation as infected states, while *S* and *R* are regarded as uninfected states, and define the system state vector as $Z={\left(S,L_{1},L_{2},C,H,R\right)}^{T}$. According to the next-generation matrix method, system (1) can be written as

(2) $$\frac{dZ}{dt}=F_{1}-V_{1},$$

where $F_{1}=\left(\begin{array}{c} 0\\ \alpha \left(\eta_{1}L_{1}+C\right)S\\ \beta \left(\eta_{2}L_{2}+C\right)S\\ 0\\ \begin{array}{c} 0\\ 0 \end{array} \end{array}\right)$, $V_{1}=\left(\begin{array}{c} -\Lambda +\alpha \left(\eta_{1}L_{1}+C\right)S+\beta \left(\eta_{2}L_{2}+C\right)S+\mu S\\ \left(\theta_{1}+\gamma +\mu \right)L_{1}\\ \left(\theta_{2}+\delta +\mu \right)L_{2}\\ \left(\sigma +\varphi +\mu \right)C-\theta_{1}L_{1}-\theta_{2}L_{2}-\psi H\\ \left(\psi +\zeta +\mu \right)H-\sigma C\\ -\gamma L_{1}-\delta L_{2}-\varphi C-\zeta H+\mu R \end{array}\right)$.

Retaining only the infected subsystem $X={\left(L_{1},L_{2},C,H\right)}^{T}$, we can write

(3) $$\frac{dX}{dt}=F-V.$$

At the rumor-free equilibrium $E_{0}$, the Jacobian matrices of F and V are computed and denoted by *F* and *V*, respectively: $F={\left.\frac{\partial F}{\partial X}\right|}_{E_{0}}=\left(\begin{array}{cccc} \alpha \eta_{1} & 0 & \alpha & 0\\ 0 & \beta \eta_{2} & \beta & 0\\ 0 & 0 & 0 & 0\\ 0 & 0 & 0 & 0 \end{array}\right)$, $V={\left.\frac{\partial V}{\partial X}\right|}_{E_{0}}=\left(\begin{array}{cccc} A_{1} & 0 & 0 & 0\\ 0 & A_{2} & 0 & 0\\ -\theta_{1} & -\theta_{2} & B & -\psi \\ 0 & 0 & -\sigma & D_{H} \end{array}\right)$, where B=σ+φ+μ, $D_{H}=\psi +\zeta +\mu$.

Therefore, the basic reproduction number is

$$R_{0}=\rho \left(FV^{-1}\right)=\frac{1}{2}\left[\Phi_{1}+\Phi_{2}+\sqrt{{\left(\Phi_{1}-\Phi_{2}\right)}^{2}+\frac{4\alpha \beta \theta_{1}\theta_{2}}{A_{1}A_{2}k_{C}^{2}}}\right]$$

where $\Phi_{1}=\frac{\alpha }{A_{1}}\left(\eta_{1}+\frac{\theta_{1}}{k_{C}}\right)$, $\;\Phi_{2}=\frac{\beta }{A_{2}}\left(\eta_{2}+\frac{\theta_{2}}{k_{C}}\right)$.

To improve readability, the main derived composite parameters and constants used in the subsequent threshold and stability analysis are summarized in Table 2.

### 3.2. Stability of the Rumor-Free Equilibrium

To further reveal the threshold mechanism and long-term dynamical evolution of bilingual coupled rumor propagation under the effect of delay, we first investigate the stability of the rumor-free equilibrium $E_{0}$ of system (1) in the case τ=0.

**Theorem 1.**  *When τ=0 and $R_{0}\leq 1$, the rumor-free equilibrium is globally asymptotically stable.*

**Proof of Theorem 1.**  Since the evolution of rumor propagation is mainly determined by the infected variables $L_{1}$, $L_{2}$, *C*, and *H* of system (1), to prove the global asymptotic stability of $E_{0}$ when τ=0 and $R_{0}\leq 1$, we construct the following Lyapunov function based on the infected variables, by means of the Lyapunov direct method and LaSalle’s invariance principle:

(4) $$V\left(t\right)=k_{1}L_{1}\left(t\right)+k_{2}L_{2}\left(t\right)+k_{3}C\left(t\right)+k_{4}H\left(t\right),$$

Since $V^{-1}F$ is a nonnegative matrix, it follows from the Perron–Frobenius theorem that there exists a positive eigenvector w>0 such that ${\left(V^{-1}F\right)}^{T}w=R_{0}w$. Let $k=V^{-T}w$. Then, $k^{T}\left(V^{-1}F\right)=R_{0}k^{T}$, and hence $k^{T}F=R_{0}k^{T}V$, which implies $k^{T}\left(F-V\right)X=\left(R_{0}-1\right)k^{T}VX$. Meanwhile, combining $k=V^{-T}w$ with $\Delta =BD_{H}-\sigma \psi >0$, we obtain $k_{3}=\frac{D_{H}K}{\Delta }$, $k_{4}=\frac{\psi K}{\Delta }$, where $K=\alpha k_{1}+\beta k_{2}$. Clearly, in the feasible region, V(t)≥0, and V(t)=0 if and only if $L_{1}=L_{2}=C=H=0$.

Differentiating V(t) and substituting $\frac{dL_{1}}{dt}$, $\frac{dL_{2}}{dt}$, $\frac{dC}{dt}$, and $\frac{dH}{dt}$ from system (1) with τ=0, we obtain

$$\begin{array}{cc} \dot{V}\left(t\right) & =\left(k_{1}\alpha \eta_{1}S\left(t\right)-k_{1}A_{1}+k_{3}\theta_{1}\right)L_{1}\left(t\right)+\left(k_{2}\beta \eta_{2}S\left(t\right)-k_{2}A_{2}+k_{3}\theta_{2}\right)L_{2}\left(t\right)\\ \; & \;+\left(k_{1}\alpha S\left(t\right)+k_{2}\beta S\left(t\right)-k_{3}B+k_{4}\sigma \right)C\left(t\right)+\left(k_{3}\psi -k_{4}D_{H}\right)H\left(t\right)\\ \; & \leq k_{1}\left(\alpha \eta_{1}-A_{1}\right)L_{1}\left(t\right)+k_{2}\left(\beta \eta_{2}-A_{2}\right)L_{2}\left(t\right)+k_{3}\theta_{1}L_{1}\left(t\right)+k_{3}\theta_{2}L_{2}\left(t\right)\\ \; & \leq \left(R_{0}-1\right)\left(k_{1}A_{1}L_{1}\left(t\right)+k_{2}A_{2}L_{2}\left(t\right)\right), \end{array}$$

Therefore, when $R_{0}\leq 1$, V(t) is monotonically nonincreasing. Moreover, when $\dot{V}\left(t\right)=0$, we have $L_{1}\left(t\right)=L_{2}\left(t\right)=0$, while $\left(C(t),H(t)\right)$ reduces to a linear system. Under the condition $\Delta =BD_{H}-\sigma \psi >0$, it follows that $\left(C(t),H(t))\to (0,0\right)$. Hence, the largest invariant set in $\dot{V}=0$ consists only of the equilibrium $E_{0}$. By LaSalle’s invariance principle, for any feasible initial condition, the solution of the system eventually converges to $E_{0}$, that is, $E_{0}$ is globally asymptotically stable. □

### 3.3. Stability of the Rumor-Prevailing Equilibrium and Hopf Bifurcation

Next, we further discuss the stability of the rumor-prevailing equilibrium $E_{1}=\left(S^{*},L_{1}^{*},L_{2}^{*},C^{*},H^{*},R^{*}\right)$.

**Theorem 2.**  *Under the Hopf bifurcation conditions, the rumor-prevailing equilibrium $E_{1}$ is locally asymptotically stable for $0\leq \tau <\tau_{0}$. When $\tau =\tau_{0}$, a pair of characteristic roots crosses the imaginary axis, $E_{1}$ loses local stability, and a local Hopf bifurcation occurs. For τ slightly larger than $\tau_{0}$, small-amplitude periodic oscillations may emerge near $E_{1}$.*

**Proof of Theorem 2.**  When τ≠0, by linearizing system (1) at the rumor-prevailing equilibrium $E_{1}=\left(S^{*},L_{1}^{*},L_{2}^{*},C^{*},H^{*},R^{*}\right)$, we obtain the corresponding characteristic matrix M(λ,τ). Let $det\left(M\left(\lambda ,\tau \right)\right)=0$. Then, the characteristic equation of the linearized system of the S2LCHR model at the rumor-prevailing equilibrium is obtained. The matrix M(λ,τ) and the characteristic equation are given as follows:

(5) $$M\left(\lambda ,\tau \right)=\left(\begin{array}{cccccc} \lambda +m_{11} & m_{12} & m_{13} & m_{14} & 0 & 0\\ -m_{21} & \lambda -m_{22}+\theta_{1}e^{-\lambda \tau } & 0 & -m_{24} & 0 & 0\\ -m_{31} & 0 & \lambda -m_{33}+\theta_{2}e^{-\lambda \tau } & -m_{34} & 0 & 0\\ 0 & -\theta_{1}e^{-\lambda \tau } & -\theta_{2}e^{-\lambda \tau } & \lambda +m_{44} & -m_{45} & 0\\ 0 & 0 & 0 & -m_{54} & \lambda +m_{55} & 0\\ 0 & -m_{62} & -m_{63} & -m_{64} & -m_{65} & \lambda +m_{66} \end{array}\right),$$

where $m_{11}=\alpha \eta_{1}L_{1}^{*}+\beta \eta_{2}L_{2}^{*}+\left(\alpha +\beta \right)C^{*}+\mu$, $m_{12}=\alpha \eta_{1}S^{*}$, $m_{13}=\beta \eta_{2}S^{*}$, $m_{14}=\left(\alpha +\beta \right)S^{*}$, $m_{21}=\alpha \left(\eta_{1}L_{1}^{*}+C^{*}\right)$, $m_{22}=\alpha \eta_{1}S^{*}-\left(\gamma +\mu \right)$, $m_{24}=\alpha S^{*}$, $m_{31}=\beta \left(\eta_{2}L_{2}^{*}+C^{*}\right)$, $m_{33}=\beta \eta_{2}S^{*}-\left(\delta +\mu \right)$, $m_{34}=\beta S^{*}$, $m_{44}=\sigma +\varphi +\mu$, $m_{45}=\psi$, $m_{54}=\sigma$, $m_{55}=\psi +\zeta +\mu$, $m_{62}=\gamma$, $m_{63}=\delta$, $m_{64}=\varphi$, $m_{65}=\zeta$, $m_{66}=\mu$.

(6) $$P\left(\lambda \right)+Q\left(\lambda \right)e^{-\lambda \tau }+W\left(\lambda \right)e^{-2\lambda \tau }=0,$$

where

$$\begin{array}{c} P\left(\lambda \right)=\lambda^{6}+a_{5}\lambda^{5}+a_{4}\lambda^{4}+a_{3}\lambda^{3}+a_{2}\lambda^{2}+a_{1}\lambda +a_{0}, \end{array}$$

$$\begin{array}{c} Q\left(\lambda \right)=b_{5}\lambda^{5}+b_{4}\lambda^{4}+b_{3}\lambda^{3}+b_{2}\lambda^{2}+b_{1}\lambda +b_{0}, \end{array}$$

$$\begin{array}{c} W\left(\lambda \right)=c_{4}\lambda^{4}+c_{3}\lambda^{3}+c_{2}\lambda^{2}+c_{1}\lambda +c_{0}, \end{array}$$

$$a_{5}=m_{11}-m_{12}-m_{13}+m_{44}+m_{55}+m_{66},$$

$$\begin{array}{c} a_{4}=-m_{11}m_{22}-m_{11}m_{33}+m_{11}m_{44}+m_{11}m_{55}+m_{11}m_{66}+m_{12}m_{21}+m_{13}m_{31}+m_{22}m_{33}-m_{22}m_{44}-m_{22}m_{55}-m_{22}m_{66}-m_{33}m_{44}\\ \;-m_{33}m_{55}-m_{33}m_{66}+m_{44}m_{55}+m_{44}m_{66}-m_{45}m_{54}+m_{55}m_{66}, \end{array}$$

$$\begin{array}{c} a_{3}=m_{11}m_{22}m_{33}-m_{11}m_{22}m_{44}-m_{11}m_{22}m_{55}-m_{11}m_{22}m_{66}-m_{11}m_{33}m_{44}-m_{11}m_{33}m_{55}-m_{11}m_{33}m_{66}+m_{11}m_{44}m_{55}+m_{11}m_{44}m_{66}\\ \;-m_{11}m_{45}m_{54}+m_{11}m_{55}m_{66}-m_{12}m_{21}m_{33}+m_{12}m_{21}m_{44}+m_{12}m_{21}m_{55}+m_{12}m_{21}m_{66}-m_{13}m_{22}m_{31}+m_{13}m_{31}m_{44}+m_{13}m_{31}m_{55}\\ \;+m_{13}m_{31}m_{66}+m_{22}m_{33}m_{44}+m_{22}m_{33}m_{55}+m_{22}m_{33}m_{66}-m_{22}m_{44}m_{55}-m_{22}m_{44}m_{66}+m_{22}m_{45}m_{54}-m_{22}m_{55}m_{66}-m_{33}m_{44}m_{55}\\ \;-m_{33}m_{44}m_{66}+m_{33}m_{45}m_{54}-m_{33}m_{55}m_{66}+m_{44}m_{55}m_{66}-m_{45}m_{54}m_{66}, \end{array}$$

$$\begin{array}{c} a_{2}=m_{11}m_{22}m_{33}m_{44}+m_{11}m_{22}m_{33}m_{55}+m_{11}m_{22}m_{33}m_{66}-m_{11}m_{22}m_{44}m_{55}-m_{11}m_{22}m_{44}m_{66}+m_{11}m_{22}m_{45}m_{54}-m_{11}m_{22}m_{55}m_{66}\\ \;-m_{11}m_{33}m_{44}m_{55}-m_{11}m_{33}m_{44}m_{66}+m_{11}m_{33}m_{45}m_{54}-m_{11}m_{33}m_{55}m_{66}+m_{11}m_{44}m_{55}m_{66}-m_{11}m_{45}m_{54}m_{66}-m_{12}m_{21}m_{33}m_{44}\\ \;-m_{12}m_{21}m_{33}m_{55}-m_{12}m_{21}m_{33}m_{66}+m_{12}m_{21}m_{44}m_{55}+m_{12}m_{21}m_{44}m_{66}-m_{12}m_{21}m_{45}m_{54}+m_{12}m_{21}m_{55}m_{66}-m_{13}m_{22}m_{31}m_{44}\\ \;-m_{13}m_{22}m_{31}m_{55}-m_{13}m_{22}m_{31}m_{66}+m_{13}m_{31}m_{44}m_{55}+m_{13}m_{31}m_{44}m_{66}-m_{13}m_{31}m_{45}m_{54}+m_{13}m_{31}m_{55}m_{66}+m_{22}m_{33}m_{44}m_{55}\\ \;+m_{22}m_{33}m_{44}m_{66}-m_{22}m_{33}m_{45}m_{54}+m_{22}m_{33}m_{55}m_{66}-m_{22}m_{44}m_{55}m_{66}+m_{22}m_{45}m_{54}m_{66}-m_{33}m_{44}m_{55}m_{66}+m_{33}m_{45}m_{54}m_{66}, \end{array}$$

$$\begin{array}{c} a_{1}=m_{11}m_{22}m_{33}m_{44}m_{55}+m_{11}m_{22}m_{33}m_{44}m_{66}-m_{11}m_{22}m_{33}m_{45}m_{54}+m_{11}m_{22}m_{33}m_{55}m_{66}-m_{11}m_{22}m_{44}m_{55}m_{66}+m_{11}m_{22}m_{45}m_{54}m_{66}\\ \;-m_{11}m_{33}m_{44}m_{55}m_{66}+m_{11}m_{33}m_{45}m_{54}m_{66}-m_{12}m_{21}m_{33}m_{44}m_{55}-m_{12}m_{21}m_{33}m_{44}m_{66}+m_{12}m_{21}m_{33}m_{45}m_{54}-m_{12}m_{21}m_{33}m_{55}m_{66}\\ \;+m_{12}m_{21}m_{44}m_{55}m_{66}-m_{12}m_{21}m_{45}m_{54}m_{66}-m_{13}m_{22}m_{31}m_{44}m_{55}-m_{13}m_{22}m_{31}m_{44}m_{66}+m_{13}m_{22}m_{31}m_{45}m_{54}-m_{13}m_{22}m_{31}m_{55}m_{66}\\ \;+m_{13}m_{31}m_{44}m_{55}m_{66}-m_{13}m_{31}m_{45}m_{54}m_{66}+m_{22}m_{33}m_{44}m_{55}m_{66}-m_{22}m_{33}m_{45}m_{54}m_{66}, \end{array}$$

$$\begin{array}{c} a_{0}=m_{11}m_{22}m_{33}m_{44}m_{55}m_{66}-m_{11}m_{22}m_{33}m_{45}m_{54}m_{66}-m_{12}m_{21}m_{33}m_{44}m_{55}m_{66}+m_{12}m_{21}m_{33}m_{45}m_{54}m_{66}-m_{13}m_{22}m_{31}m_{44}m_{55}m_{66}\\ \;+m_{13}m_{22}m_{31}m_{45}m_{54}m_{66}, \end{array}$$

$$\begin{array}{c} b_{5}=\theta_{1}+\theta_{2}, \end{array}$$

$$\begin{array}{c} b_{4}=m_{11}\theta_{1}+m_{11}\theta_{2}-m_{22}\theta_{2}-m_{24}\theta_{1}-m_{33}\theta_{1}-m_{34}\theta_{2}+m_{44}\theta_{1}+m_{44}\theta_{2}+m_{55}\theta_{1}+m_{55}\theta_{2}+m_{66}\theta_{1}+m_{66}\theta_{2}, \end{array}$$

$$\begin{array}{c} b_{3}=-m_{11}m_{22}\theta_{2}-m_{11}m_{24}\theta_{1}-m_{11}m_{33}\theta_{1}-m_{11}m_{34}\theta_{2}+m_{11}m_{44}\theta_{1}+m_{11}m_{44}\theta_{2}+m_{11}m_{55}\theta_{1}+m_{11}m_{55}\theta_{2}+m_{11}m_{66}\theta_{1}+m_{11}m_{66}\theta_{2}\\ \;+m_{12}m_{21}\theta_{2}+m_{13}m_{31}\theta_{1}+m_{14}m_{21}\theta_{1}+m_{14}m_{31}\theta_{2}+m_{22}m_{34}\theta_{2}-m_{22}m_{44}\theta_{2}-m_{22}m_{55}\theta_{2}-m_{22}m_{66}\theta_{2}+m_{24}m_{33}\theta_{1}-m_{24}m_{55}\theta_{1}\\ \;-m_{24}m_{66}\theta_{1}-m_{33}m_{44}\theta_{1}-m_{33}m_{55}\theta_{1}-m_{33}m_{66}\theta_{1}-m_{34}m_{55}\theta_{2}-m_{34}m_{66}\theta_{2}+m_{44}m_{55}\theta_{1}+m_{44}m_{55}\theta_{2}+m_{44}m_{66}\theta_{1}+m_{44}m_{66}\theta_{2}\\ \;-m_{45}m_{54}\theta_{1}-m_{45}m_{54}\theta_{2}+m_{55}m_{66}\theta_{1}+m_{55}m_{66}\theta_{2}, \end{array}$$

$$\begin{array}{c} b_{2}=m_{11}m_{22}m_{34}\theta_{2}-m_{11}m_{22}m_{44}\theta_{2}-m_{11}m_{22}m_{55}\theta_{2}-m_{11}m_{22}m_{66}\theta_{2}+m_{11}m_{24}m_{33}\theta_{1}-m_{11}m_{24}m_{55}\theta_{1}-m_{11}m_{24}m_{66}\theta_{1}-m_{11}m_{33}m_{44}\theta_{1}\\ \;-m_{11}m_{33}m_{55}\theta_{1}-m_{11}m_{33}m_{66}\theta_{1}-m_{11}m_{34}m_{55}\theta_{2}-m_{11}m_{34}m_{66}\theta_{2}+m_{11}m_{44}m_{55}\theta_{1}+m_{11}m_{44}m_{55}\theta_{2}+m_{11}m_{44}m_{66}\theta_{1}+m_{11}m_{44}m_{66}\theta_{2}\\ \;-m_{11}m_{45}m_{54}\theta_{1}-m_{11}m_{45}m_{54}\theta_{2}+m_{11}m_{55}m_{66}\theta_{1}+m_{11}m_{55}m_{66}\theta_{2}-m_{12}m_{21}m_{34}\theta_{2}+m_{12}m_{21}m_{44}\theta_{2}+m_{12}m_{21}m_{55}\theta_{2}+m_{12}m_{21}m_{66}\theta_{2}\\ \;+m_{12}m_{24}m_{31}\theta_{2}+m_{13}m_{21}m_{34}\theta_{1}-m_{13}m_{24}m_{31}\theta_{1}+m_{13}m_{31}m_{44}\theta_{1}+m_{13}m_{31}m_{55}\theta_{1}+m_{13}m_{31}m_{66}\theta_{1}-m_{14}m_{21}m_{33}\theta_{1}+m_{14}m_{21}m_{55}\theta_{1}\\ \;+m_{14}m_{21}m_{66}\theta_{1}-m_{14}m_{22}m_{31}\theta_{2}+m_{14}m_{31}m_{55}\theta_{2}+m_{14}m_{31}m_{66}\theta_{2}+m_{22}m_{34}m_{55}\theta_{2}+m_{22}m_{34}m_{66}\theta_{2}-m_{22}m_{44}m_{55}\theta_{2}-m_{22}m_{44}m_{66}\theta_{2}\\ \;+m_{22}m_{45}m_{54}\theta_{2}-m_{22}m_{55}m_{66}\theta_{2}+m_{24}m_{33}m_{55}\theta_{1}+m_{24}m_{33}m_{66}\theta_{1}-m_{24}m_{55}m_{66}\theta_{1}-m_{33}m_{44}m_{55}\theta_{1}-m_{33}m_{44}m_{66}\theta_{1}+m_{33}m_{45}m_{54}\theta_{1}\\ \;-m_{33}m_{55}m_{66}\theta_{1}-m_{34}m_{55}m_{66}\theta_{1}+m_{44}m_{55}m_{66}\theta_{1}+m_{44}m_{55}m_{66}\theta_{2}-m_{45}m_{54}m_{66}\theta_{1}-m_{45}m_{54}m_{66}\theta_{2}, \end{array}$$

$$\begin{array}{c} b_{1}=m_{11}m_{22}m_{34}m_{55}\theta_{2}+m_{11}m_{22}m_{34}m_{66}\theta_{2}-m_{11}m_{22}m_{44}m_{55}\theta_{2}-m_{11}m_{22}m_{44}m_{66}\theta_{2}+m_{11}m_{22}m_{45}m_{54}\theta_{2}-m_{11}m_{22}m_{55}m_{66}\theta_{2}\\ \;+m_{11}m_{24}m_{33}m_{55}\theta_{1}+m_{11}m_{24}m_{33}m_{66}\theta_{1}-m_{11}m_{24}m_{55}m_{66}\theta_{1}-m_{11}m_{33}m_{44}m_{55}\theta_{1}-m_{11}m_{33}m_{44}m_{66}\theta_{1}+m_{11}m_{33}m_{45}m_{54}\theta_{1}\\ \;-m_{11}m_{33}m_{55}m_{66}\theta_{1}-m_{11}m_{34}m_{55}m_{66}\theta_{2}+m_{11}m_{44}m_{55}m_{66}\theta_{1}+m_{11}m_{44}m_{55}m_{66}\theta_{2}-m_{11}m_{45}m_{54}m_{66}\theta_{1}-m_{11}m_{45}m_{54}m_{66}\theta_{2}\\ \;-m_{12}m_{21}m_{34}m_{55}\theta_{2}-m_{12}m_{21}m_{34}m_{66}\theta_{2}+m_{12}m_{21}m_{44}m_{55}\theta_{2}+m_{12}m_{21}m_{44}m_{66}\theta_{2}-m_{12}m_{21}m_{45}m_{54}\theta_{2}+m_{12}m_{21}m_{55}m_{66}\theta_{2}\\ \;+m_{12}m_{24}m_{31}m_{55}\theta_{2}+m_{12}m_{24}m_{31}m_{66}\theta_{2}+m_{13}m_{21}m_{34}m_{55}\theta_{1}+m_{13}m_{21}m_{34}m_{66}\theta_{1}-m_{13}m_{24}m_{31}m_{55}\theta_{1}-m_{13}m_{24}m_{31}m_{66}\theta_{1}\\ \;+m_{13}m_{31}m_{44}m_{55}\theta_{1}+m_{13}m_{31}m_{44}m_{66}\theta_{1}-m_{13}m_{31}m_{45}m_{54}\theta_{1}+m_{13}m_{31}m_{55}m_{66}\theta_{1}-m_{14}m_{21}m_{33}m_{55}\theta_{1}-m_{14}m_{21}m_{33}m_{66}\theta_{1}\\ \;+m_{14}m_{21}m_{55}m_{66}\theta_{1}-m_{14}m_{22}m_{31}m_{55}\theta_{2}-m_{14}m_{22}m_{31}m_{66}\theta_{2}+m_{14}m_{31}m_{55}m_{66}\theta_{2}+m_{22}m_{34}m_{55}m_{66}\theta_{2}-m_{22}m_{44}m_{55}m_{66}\theta_{2}\\ \;+m_{22}m_{45}m_{54}m_{66}\theta_{2}+m_{24}m_{33}m_{55}m_{66}\theta_{1}-m_{33}m_{44}m_{55}m_{66}\theta_{1}+m_{33}m_{45}m_{54}m_{66}\theta_{1}, \end{array}$$

$$\begin{array}{c} b_{0}=m_{11}m_{22}m_{34}m_{55}m_{66}\theta_{2}-m_{11}m_{22}m_{44}m_{55}m_{66}\theta_{2}+m_{11}m_{22}m_{45}m_{54}m_{66}\theta_{2}+m_{11}m_{24}m_{33}m_{55}m_{66}\theta_{1}-m_{11}m_{33}m_{44}m_{55}m_{66}\theta_{1}\\ \;+m_{11}m_{33}m_{45}m_{54}m_{66}\theta_{1}-m_{12}m_{21}m_{34}m_{55}m_{66}\theta_{2}+m_{12}m_{21}m_{44}m_{55}m_{66}\theta_{2}-m_{12}m_{21}m_{45}m_{54}m_{66}\theta_{2}+m_{12}m_{24}m_{31}m_{55}m_{66}\theta_{2}\\ \;+m_{13}m_{21}m_{34}m_{55}m_{66}\theta_{1}-m_{13}m_{24}m_{31}m_{55}m_{66}\theta_{1}+m_{13}m_{31}m_{44}m_{55}m_{66}\theta_{1}-m_{13}m_{31}m_{45}m_{54}m_{66}\theta_{1}-m_{14}m_{21}m_{33}m_{55}m_{66}\theta_{1}\\ \;-m_{14}m_{22}m_{31}m_{55}m_{66}\theta_{2}, \end{array}$$

$$\begin{array}{c} c_{4}=\theta_{1}\theta_{2}, \end{array}$$

$$\begin{array}{c} c_{3}=m_{11}\theta_{1}\theta_{2}-m_{24}\theta_{1}\theta_{2}-m_{34}\theta_{1}\theta_{2}+m_{44}\theta_{1}\theta_{2}+m_{55}\theta_{1}\theta_{2}+m_{66}\theta_{1}\theta_{2}, \end{array}$$

$$\begin{array}{c} c_{2}=-m_{11}m_{24}\theta_{1}\theta_{2}-m_{11}m_{34}\theta_{1}\theta_{2}+m_{11}m_{44}\theta_{1}\theta_{2}+m_{11}m_{55}\theta_{1}\theta_{2}+m_{11}m_{66}\theta_{1}\theta_{2}+m_{14}m_{21}\theta_{1}\theta_{2}+m_{14}m_{31}\theta_{1}\theta_{2}-m_{24}m_{55}\theta_{1}\theta_{2}\\ \;-m_{24}m_{66}\theta_{1}\theta_{2}-m_{34}m_{55}\theta_{1}\theta_{2}-m_{34}m_{66}\theta_{1}\theta_{2}+m_{44}m_{55}\theta_{1}\theta_{2}+m_{44}m_{66}\theta_{1}\theta_{2}-m_{45}m_{54}\theta_{1}\theta_{2}+m_{55}m_{66}\theta_{1}\theta_{2}, \end{array}$$

$$\begin{array}{c} c_{1}=-m_{11}m_{24}m_{55}\theta_{1}\theta_{2}-m_{11}m_{24}m_{66}\theta_{1}\theta_{2}-m_{11}m_{34}m_{55}\theta_{1}\theta_{2}-m_{11}m_{34}m_{66}\theta_{1}\theta_{2}+m_{11}m_{44}m_{55}\theta_{1}\theta_{2}+m_{11}m_{44}m_{66}\theta_{1}\theta_{2}\\ \;-m_{11}m_{45}m_{54}\theta_{1}\theta_{2}+m_{11}m_{55}m_{66}\theta_{1}\theta_{2}+m_{14}m_{21}m_{55}\theta_{1}\theta_{2}+m_{14}m_{21}m_{66}\theta_{1}\theta_{2}+m_{14}m_{31}m_{55}\theta_{1}\theta_{2}+m_{14}m_{31}m_{66}\theta_{1}\theta_{2}\\ \;-m_{24}m_{55}m_{66}\theta_{1}\theta_{2}-m_{34}m_{55}m_{66}\theta_{1}\theta_{2}+m_{44}m_{55}m_{66}\theta_{1}\theta_{2}-m_{45}m_{54}m_{66}\theta_{1}\theta_{2}, \end{array}$$

$$\begin{array}{c} c_{0}=-m_{11}m_{24}m_{55}m_{66}\theta_{1}\theta_{2}-m_{11}m_{34}m_{55}m_{66}\theta_{1}\theta_{2}+m_{11}m_{44}m_{55}m_{66}\theta_{1}\theta_{2}-m_{11}m_{45}m_{54}m_{66}\theta_{1}\theta_{2}+m_{14}m_{21}m_{55}m_{66}\theta_{1}\theta_{2}\\ \;+m_{14}m_{31}m_{55}m_{66}\theta_{1}\theta_{2}. \end{array}$$

Therefore, when τ=0, we can obtain the characteristic equation of the linearized part of system (1) at $E_{1}$ as

(7) $$\lambda^{6}+B_{5}\lambda^{5}+B_{4}\lambda^{4}+B_{3}\lambda^{3}+B_{2}\lambda^{2}+B_{1}\lambda +B_{0}=0,$$

where $B_{i}=a_{i}+b_{i}+c_{i}$, i=0,1,2,3,4,5.

**Inference 1.**  The following conditions hold:

$\Delta_{1}=B_{5}>0$, $\Delta_{2}=\left|\begin{array}{cc} B_{5} & B_{3}\\ 1 & B_{4} \end{array}\right|>0$, $\Delta_{3}=\left|\begin{array}{ccc} B_{5} & B_{3} & B_{1}\\ 1 & B_{4} & B_{2}\\ 0 & B_{5} & B_{3} \end{array}\right|>0$,

$\Delta_{4}=\left|\begin{array}{cccc} B_{5} & B_{3} & B_{1} & 0\\ 1 & B_{4} & B_{2} & B_{0}\\ 0 & B_{5} & B_{3} & B_{1}\\ 0 & 1 & B_{4} & B_{2} \end{array}\right|>0$, $\Delta_{5}=\left|\begin{array}{ccccc} B_{5} & B_{3} & B_{1} & 0 & 0\\ 1 & B_{4} & B_{2} & B_{0} & 0\\ 0 & B_{5} & B_{3} & B_{1} & 0\\ 0 & 1 & B_{4} & B_{2} & B_{0}\\ 0 & 0 & B_{5} & B_{3} & B_{1} \end{array}\right|>0$,

$\Delta_{6}=\left|\begin{array}{cccccc} B_{5} & B_{3} & B_{1} & 0 & 0 & 0\\ 1 & B_{4} & B_{2} & B_{0} & 0 & 0\\ 0 & B_{5} & B_{3} & B_{1} & 0 & 0\\ 0 & 1 & B_{4} & B_{2} & B_{0} & 0\\ 0 & 0 & B_{5} & B_{3} & B_{1} & 0\\ 0 & 0 & 1 & B_{4} & B_{2} & B_{0} \end{array}\right|>0$.

The determinants $\Delta_{1},\ldots ,\Delta_{6}$ are general Routh–Hurwitz conditions for the sixth-degree characteristic polynomial obtained from the linearized system at $E_{1}$ when τ=0. For a specific parameter set, the coefficients $B_{i}$ and the determinants $\Delta_{i}$ must be evaluated after substituting the corresponding rumor-prevailing equilibrium $E_{1}$. To make this verification transparent, the numerical values of $\Delta_{1},\ldots ,\Delta_{6}$ for the main $R_{0}>1$ parameter sets used in the simulations are reported in Appendix B, Table A1.

When the above Routh-Hurwitz conditions hold, all roots of the characteristic equation have negative real parts. Therefore, the rumor-prevailing equilibrium $E_{1}$ is locally asymptotically stable. This indicates that, under the current parameter conditions, the coupled rumor propagation system in a bilingual environment gradually returns to its original stable propagation state after small perturbations, rather than exhibiting sustained amplification or severe fluctuations. From the perspective of practical propagation, the proportions of users in states $L_{1}$, $L_{2}$, *C*, and *H* eventually remain relatively stable within a certain range. This implies that although rumors may still persist in bilingual OSNs, their cross-lingual coupled propagation does not easily evolve into a sudden large-scale outbreak, thereby keeping the public risks caused by a rapid escalation of public opinion within a relatively controllable range.

When τ>0, to further analyze the effect of delay on the stability of the system, let λ=iω(ω>0) be a purely imaginary root of the characteristic equation. Substituting it into the characteristic equation and separating the real and imaginary parts, we obtain the following system of equations:

(8) $$\left\{\begin{array}{c} P_{R}\left(\omega \right)+Q_{R}\left(\omega \right)\cos\left(\omega \tau \right)+Q_{I}\left(\omega \right)\sin\left(\omega \tau \right)+W_{R}\left(\omega \right)\cos\left(2\omega \tau \right)+W_{I}\left(\omega \right)\sin\left(2\omega \tau \right)=0,\\ P_{I}\left(\omega \right)+Q_{I}\left(\omega \right)\cos\left(\omega \tau \right)-Q_{R}\left(\omega \right)\sin\left(\omega \tau \right)+W_{I}\left(\omega \right)\cos\left(2\omega \tau \right)-W_{R}\left(\omega \right)\sin\left(2\omega \tau \right)=0, \end{array}\right.$$

in which,

$P_{R}\left(\omega \right)=-\omega^{6}+a_{4}\omega^{4}-a_{2}\omega^{2}+a_{0}$, $P_{I}\left(\omega \right)=a_{5}\omega^{5}-a_{3}\omega^{3}+a_{1}\omega$,

$Q_{R}\left(\omega \right)=b_{4}\omega^{4}-b_{2}\omega^{2}+b_{0}$, $Q_{I}\left(\omega \right)=b_{5}\omega^{5}-b_{3}\omega^{3}+b_{1}\omega$,

$W_{R}\left(\omega \right)=c_{4}\omega^{4}-c_{2}\omega^{2}+c_{0}$, $W_{I}\left(\omega \right)=-c_{3}\omega^{3}+c_{1}\omega$.

Squaring both sides of the above two equations and then adding them together eliminates the related trigonometric terms, thereby yielding an algebraic equation involving only ω:

(9) $$N{\left(\omega \right)}^{2}-D_{R}{\left(\omega \right)}^{2}-D_{I}{\left(\omega \right)}^{2}=0,$$

where,

$N\left(\omega \right)=\left(W_{R}{\left(\omega \right)}^{2}+W_{I}{\left(\omega \right)}^{2}\right)-\left(P_{R}{\left(\omega \right)}^{2}+P_{I}{\left(\omega \right)}^{2}\right)$,

$D_{R}\left(\omega \right)=\left(P_{R}\left(\omega \right)Q_{R}\left(\omega \right)+P_{I}\left(\omega \right)Q_{I}\left(\omega \right)\right)-\left(W_{R}\left(\omega \right)Q_{R}\left(\omega \right)+W_{I}\left(\omega \right)Q_{I}\left(\omega \right)\right)$,

$D_{I}\left(\omega \right)=\left(P_{R}\left(\omega \right)+W_{R}\left(\omega \right)\right)Q_{I}\left(\omega \right)-\left(P_{I}\left(\omega \right)+W_{I}\left(\omega \right)\right)Q_{R}\left(\omega \right)$.

**Inference 2.**  Let $G\left(\omega \right)=N{\left(\omega \right)}^{2}-D_{R}{\left(\omega \right)}^{2}-D_{I}{\left(\omega \right)}^{2}$. If there exists $\omega_{0}>0$ such that $G\left(\omega_{0}\right)=0,G^{\prime }\left(\omega_{0}\right)\neq 0$, then $\omega_{0}$ is a simple positive root of G(ω)=0. Therefore, the characteristic equation admits a pair of simple purely imaginary roots $\lambda =\pm i\omega_{0}$ at the corresponding critical delay $\tau_{0}$.

Therefore, the above result satisfies the necessary condition for the characteristic equation to admit a pair of purely imaginary roots. By further calculation, we obtain the following equation.

(10) $$\tau_{j}=\frac{1}{\omega_{0}}\left[\arccos\left(\frac{N\left(\omega_{0}\right)D_{R}\left(\omega_{0}\right)}{D_{R}{\left(\omega_{0}\right)}^{2}+D_{I}{\left(\omega_{0}\right)}^{2}}\right)+2j\pi \right],$$

where j=0,1,2,…, and define the minimum positive critical value as $\tau_{0}=\min_{j\geq 0}\tau_{j}$.

To further characterize how the characteristic roots vary with the parameter, we differentiate Equation (6) with respect to λ, which yields

(11) $${\left(\frac{d\lambda }{d\tau }\right)}^{-1}=\frac{P^{\prime }\left(\lambda \right)\left(Q^{\prime }\left(\lambda \right)-\tau Q\left(\lambda \right)\right)e^{-\lambda \tau }+\left(W^{\prime }\left(\lambda \right)-2\tau W\left(\lambda \right)\right)e^{-2\lambda \tau }}{\lambda Q\left(\lambda \right)e^{-\lambda \tau }+2\lambda W\left(\lambda \right)e^{-2\lambda \tau }}.$$

Furthermore, substituting λ=iω(ω>0) into Equation (11), we obtain the following expression. This result provides an important basis for the subsequent analysis of the direction in which the characteristic roots cross the imaginary axis and for verifying the transversality condition of the Hopf bifurcation.

(12) Redλdτ−1τ=τ0=Π1ω0Ω1ω0+Π2ω0Ω2ω0Ω12ω0+Ω22ω0,

where,

$$\Omega_{1}\left(\omega_{0}\right)=-\omega_{0}\left[\left(Q_{I}\left(\omega_{0}\right)\cos\left(\omega_{0}\tau_{0}\right)-Q_{R}\left(\omega_{0}\right)\sin\left(\omega_{0}\tau_{0}\right)\right)+2\left(W_{I}\left(\omega_{0}\right)\cos\left(2\omega_{0}\tau_{0}\right)-W_{R}\left(\omega_{0}\right)\sin\left(2\omega_{0}\tau_{0}\right)\right)\right],$$

$$\Omega_{2}\left(\omega_{0}\right)=\omega_{0}\left[\left(Q_{R}\left(\omega_{0}\right)\cos\left(\omega_{0}\tau_{0}\right)+Q_{I}\left(\omega_{0}\right)\sin\left(\omega_{0}\tau_{0}\right)\right)+2\left(W_{R}\left(\omega_{0}\right)\cos\left(2\omega_{0}\tau_{0}\right)+W_{I}\left(\omega_{0}\right)\sin\left(2\omega_{0}\tau_{0}\right)\right)\right],$$

$$\begin{array}{c} \Pi_{1}\left(\omega_{0}\right)={P^{\prime }}_{I}\left(\omega_{0}\right)+{Q^{\prime }}_{I}\left(\omega_{0}\right)\cos\left(\omega_{0}\tau_{0}\right)-Q_{R}\left(\omega_{0}\right)\sin\left(\omega_{0}\tau_{0}\right)+{W^{\prime }}_{I}\left(\omega_{0}\right)\cos\left(2\omega_{0}\tau_{0}\right)+{W^{\prime }}_{R}\left(\omega_{0}\right)\sin\left(2\omega_{0}\tau_{0}\right)\\ \;-\tau_{0}\left[\left(Q_{R}\left(\omega_{0}\right)\cos\left(\omega_{0}\tau_{0}\right)+Q_{I}\left(\omega_{0}\right)\sin\left(\omega_{0}\tau_{0}\right)\right)+2\left(W_{R}\left(\omega_{0}\right)\cos\left(2\omega_{0}\tau_{0}\right)+W_{I}\left(\omega_{0}\right)\sin\left(2\omega_{0}\tau_{0}\right)\right)\right], \end{array}$$

$$\begin{array}{c} \Pi_{2}\left(\omega_{0}\right)={P^{\prime }}_{R}\left(\omega_{0}\right)+{Q^{\prime }}_{R}\left(\omega_{0}\right)\cos\left(\omega_{0}\tau_{0}\right)-{Q^{\prime }}_{I}\left(\omega_{0}\right)\sin\left(\omega_{0}\tau_{0}\right)+{W^{\prime }}_{R}\left(\omega_{0}\right)\cos\left(2\omega_{0}\tau_{0}\right)-{W^{\prime }}_{I}\left(\omega_{0}\right)\sin\left(2\omega_{0}\tau_{0}\right)\\ \;-\tau_{0}\left[\left(Q_{I}\left(\omega_{0}\right)\cos\left(\omega_{0}\tau_{0}\right)-Q_{R}\left(\omega_{0}\right)\sin\left(\omega_{0}\tau_{0}\right)\right)+2\left(W_{I}\left(\omega_{0}\right)\cos\left(2\omega_{0}\tau_{0}\right)-W_{R}\left(\omega_{0}\right)\sin\left(2\omega_{0}\tau_{0}\right)\right)\right], \end{array}$$

$$P_{R}^{\prime }\left(\omega_{0}\right)=-6\omega_{0}^{5}+4a_{4}\omega_{0}^{3}-2a_{2}\omega_{0},\;\;P_{I}^{\prime }\left(\omega_{0}\right)=5a_{5}\omega_{0}^{4}-3a_{3}\omega_{0}^{2}+a_{1},\;\;Q_{R}^{\prime }\left(\omega_{0}\right)=4b_{4}\omega_{0}^{3}-2b_{2}\omega_{0},$$

$$Q_{I}^{\prime }\left(\omega_{0}\right)=5b_{5}\omega_{0}^{4}-3b_{3}\omega_{0}^{2}+b_{1},\;\;W_{R}^{\prime }\left(\omega_{0}\right)=4c_{4}\omega_{0}^{3}-2c_{2}\omega_{0},\;\;W_{I}^{\prime }\left(\omega_{0}\right)=-3c_{3}\omega_{0}^{2}+c_{1},$$

**Inference 3.**  Redλdτ−1τ=τ0,λ=iω0≠0.

For clarity, the Hopf bifurcation conditions used in this study are summarized as follows:

**I1. Delay-free stability.** When τ=0, the rumor-prevailing equilibrium $E_{1}$ is locally asymptotically stable. This condition is verified by the Routh–Hurwitz conditions in Inference 1.

**I2. Simple purely imaginary roots.** There exists $\omega_{0}>0$ such that the characteristic equation has a pair of simple purely imaginary roots $\lambda =\pm i\omega_{0}$ at $\tau =\tau_{0}$. This condition is verified by Inference 2.

**I3. Nonzero transversality.** The characteristic roots cross the imaginary axis with nonzero speed, namely, Redλdττ=τ0,λ=iω0≠0. This condition is verified by Inference 3.

Therefore, Inferences 1–3 jointly verify the Hopf bifurcation conditions I1–I3. According to the Hopf bifurcation theorem, when τ passes through $\tau_{0}$, the rumor-prevailing equilibrium $E_{1}$ loses local stability and a local Hopf bifurcation occurs. For $0\leq \tau <\tau_{0}$, $E_{1}$ remains locally asymptotically stable; for τ slightly larger than $\tau_{0}$, small-amplitude periodic oscillations may emerge near $E_{1}$. □

## 4. Numerical Simulations and Analysis

In this section, we use numerical simulations to examine the threshold role of $R_{0}$, delay-induced Hopf bifurcation, delay robustness when $R_{0}<1$, $R_{0}$-based parameter sensitivity, and social-impact indicators. In addition, a real-world aggregate trend-fitting case study is conducted using multilingual COVID-19-related tweet data to examine the empirical plausibility and trend-capturing ability of the delayed S2LCHR model.

All numerical simulations were performed in MATLAB. For delay-free cases (τ=0), the ODE system was solved using the adaptive solver ode45 with relative tolerance $10^{-8}$, absolute tolerance $10^{-10}$, and maximum step size 0.05. For delayed cases (τ>0), the DDE system was solved using MATLAB dde23 with nonnegative history functions. In the Hopf bifurcation simulations, the DDE solver was configured with relative tolerance $10^{-9}$, absolute tolerance $10^{-12}$, initial step size $10^{-3}$, maximum step size 0.02, and norm control enabled. For long-time delay-robustness simulations, we used relative tolerance $10^{-8}$, absolute tolerance $10^{-10}$, and maximum step size 0.5. Convergence was checked by halving the maximum step size and tightening the tolerances by one order of magnitude; the computed critical delay, convergence behavior, and oscillatory patterns remained unchanged at the plotted precision.

### 4.1. Threshold Dynamics of the Delay-Free System

**Figure 2:**
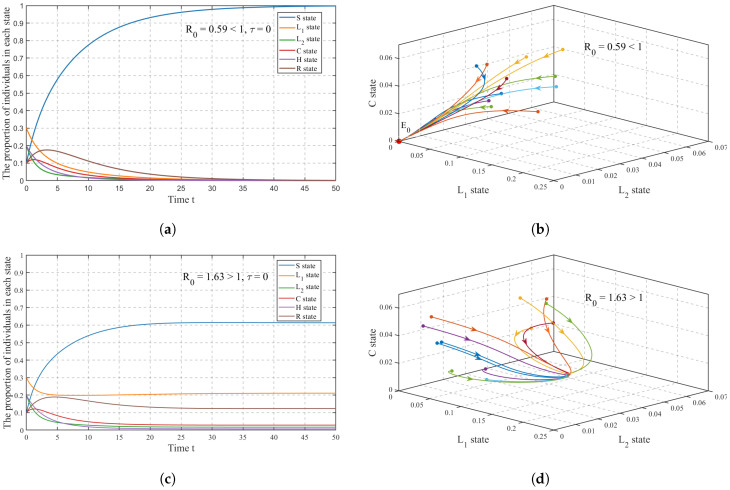
Dynamic behaviors of the system under τ=0 for $R_{0}<1$ and $R_{0}>1$: (**a**) evolution of each state for $R_{0}<1$; (**b**) convergence to $E_{0}$ for $R_{0}<1$; (**c**) evolution of each state for $R_{0}>1$; (**d**) convergence to $E_{1}$ for $R_{0}>1$.

To verify the stability results for the delay-free system established in Section 3, in this subsection we set τ=0 and discuss the long-term dynamical behaviors of the system under the two cases $R_{0}<1$ and $R_{0}>1$, respectively. Figure 2a,c present the time-series plots of each state variable under these two cases, respectively, so as to characterize the evolution of the population proportions in each state over time. Figure 2b,d show the three-dimensional phase trajectories of the system in the ($L_{1}$, $L_{2}$, *C*) space. To illustrate the convergence of system trajectories under different initial states more clearly, we randomly selected 12 dispersed groups of initial conditions within the prescribed state range for numerical simulation.

**Scenario 1.** We set the parameters as Λ=0.2, μ=0.2, α=0.2, β=0.3, $\eta_{1}=0.61$, $\eta_{2}=0.2$, $\theta_{1}=0.03$, $\theta_{2}=0.26$, σ=0.1, ψ=0.1, γ=0.09, δ=0.12, φ=0.1, and ζ=0.1, for which $R_{0}=0.58<1$. As shown in Figure 2a, when there is no delay and $R_{0}<1$, the state S(t) continues to increase over time and eventually approaches 1, whereas $L_{1}\left(t\right)$, $L_{2}\left(t\right)$, C(t), H(t), and R(t) all eventually decay to values close to 0. Among them, R(t) and C(t) exhibit brief fluctuations in the initial stage, but these gradually disappear as time evolves. This indicates that, under the current parameter settings, the rumor-related states cannot be maintained in the system in the long run, and the propagation process eventually evolves to a rumor-free state. From Figure 2b, it can be seen that although the trajectories start from different initial points and follow different evolutionary paths, they all finally converge to the same equilibrium position near $\left(L_{1},L_{2},C)=(0,0,0\right)$. This shows that when $R_{0}<1$, the solution of the system converges to the rumor-free equilibrium, and the rumor-free equilibrium is asymptotically stable in the sense of the time series. Therefore, the two figures complement each other from the perspectives of the time domain and phase space, and jointly verify the stability conclusion of Theorem 1 for the rumor-free equilibrium under the delay-free condition.

**Scenario 2.** We next consider the case $R_{0}>1$. We set the parameters as Λ=0.2, μ=0.2, α=0.7, β=0.5, $\eta_{1}=0.61$, $\eta_{2}=0.2$, $\theta_{1}=0.03$, $\theta_{2}=0.26$, σ=0.1, ψ=0.1, γ=0.09, δ=0.12, φ=0.1, and ζ=0.1, for which $R_{0}=1.63>1$. As shown in Figure 2c, when $R_{0}>1$, the state S(t) increases rapidly in the initial stage and then stabilizes around a certain positive value, while the remaining states in the system gradually approach their respective nonzero steady levels. This indicates that once $R_{0}$ exceeds the threshold value 1, the rumor does not die out naturally, but instead eventually converges to a rumor-prevailing equilibrium $E_{1}$. Furthermore, Figure 2d shows that, although the trajectories start from different initial points within the selected range of initial conditions, they all finally converge to the same nonzero equilibrium. This shows that when $R_{0}>1$, the rumor-prevailing equilibrium $E_{1}$ attracts nearby trajectories, and the system maintains a certain proportion of users in states $L_{1}$, $L_{2}$, and *C* in the long run. From the perspective of practical propagation, the rumor no longer disappears, but persists in OSNs in a relatively stable form. Therefore, the time-series results and phase trajectories corroborate each other and jointly verify the stability conclusion of Theorem 2 for the rumor-prevailing equilibrium under the delay-free condition.

Taken together, Figure 2a–d show that, under the delay-free condition, the basic reproduction number $R_{0}$ plays a clear threshold role in determining the long-term behavior of the system. This provides a numerical basis for the subsequent investigation of the system stability and Hopf bifurcation behavior under the time-delay condition.

### 4.2. Hopf Bifurcation and Periodic Behavior Analysis of the Time-Delay System

**Figure 3:**
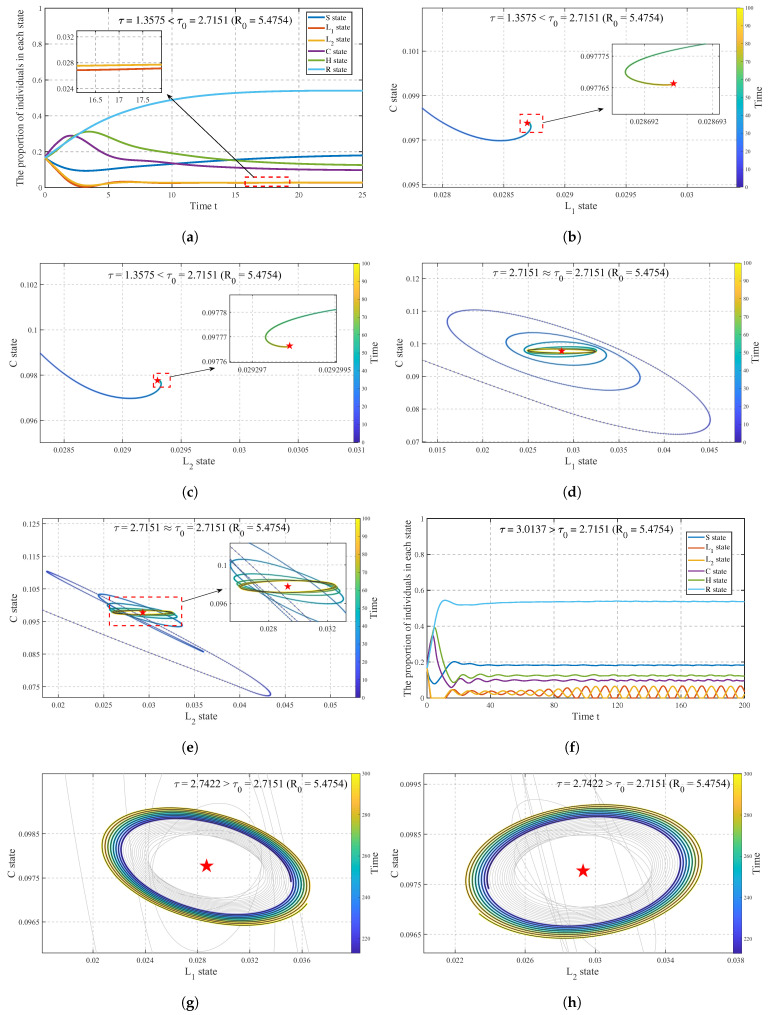
Dynamic behaviors of the delayed system around the critical delay $\tau_{0}$: (**a**) Evolution of each state for $\tau <\tau_{0}$; (**b**) $C-L_{1}$ phase portrait for $\tau <\tau_{0}$; (**c**) $C-L_{2}$ phase portrait for $\tau <\tau_{0}$; (**d**) $C-L_{1}$ phase portrait for $\tau \approx \tau_{0}$; (**e**) $C-L_{2}$ phase portrait for $\tau \approx \tau_{0}$; (**f**) Evolution of each state for $\tau >\tau_{0}$; (**g**) $C-L_{1}$ phase portrait for $\tau >\tau_{0}$; (**h**) $C-L_{2}$ phase portrait for $\tau >\tau_{0}$. The red stars in the phase portraits denote the rumor-prevailing equilibrium $E_{1}$.

In this subsection, we carry out numerical simulations around the critical delay $\tau_{0}$ to investigate the changes in the dynamical behavior of the system near the rumor-prevailing equilibrium $E_{1}$ for the cases $\tau <\tau_{0}$, $\tau \approx \tau_{0}$, and $\tau >\tau_{0}$, so as to verify the conclusions in Section 3 regarding the stability and Hopf bifurcation of the time-delay system. We set Λ=0.05, μ=0.05, α=0.95, β=0.90, $\eta_{1}=0.80$, $\eta_{2}=0.80$, $\theta_{1}=0.60$, $\theta_{2}=0.55$, σ=0.70, ψ=0.40, γ=0.08, δ=0.08, φ=0.10, and ζ=0.10, for which $R_{0}=5.48>1$. Further calculation gives the critical delay approximately as $\tau_{0}=2.7151$. Figure 3a,f present the time-series plots of each state variable for the cases $\tau <\tau_{0}$ and $\tau >\tau_{0}$, respectively, so as to characterize the evolution of the population proportions in each state over time. Figure 3b–e,g,h show the local phase trajectories of the system in the ($L_{1},C$) and ($L_{2},C$) planes under different delays, respectively, in order to observe the local dynamical properties near $E_{1}$ from the perspective of phase space.

**Scenario 1.** We first investigate the case where the delay is below the critical value. When $\tau =0.5\tau_{0}<\tau_{0}$, Figure 3a shows that, under this condition, all state variables of the system eventually approach their respective stable levels after a brief adjustment in the initial stage. This indicates that when the delay does not exceed the critical delay $\tau_{0}$, although the rumor does not disappear, the system can remain near a relatively stable rumor-prevailing equilibrium state. This result is consistent with the theoretical conclusion in Section 3 that $E_{1}$ is locally asymptotically stable when $\left.\tau \in [0,\tau_{0}\right)$. Furthermore, Figure 3b,c show that when $\tau <\tau_{0}$, although the trajectories start from different initial points, they all gradually converge to the same interior equilibrium, namely, the rumor-prevailing equilibrium $E_{1}$. From the perspective of practical propagation, this means that when the cross-lingual response delay is small, bilingual coupled rumors may persist, but their spreading scale gradually tends toward equilibrium and does not exhibit obvious periodic rebound. In summary, Figure 3a–c complement each other from the perspectives of the time domain and phase space, and jointly verify the theoretical conclusion in Theorem 2 that the rumor-prevailing equilibrium $E_{1}$ is locally asymptotically stable when $\left.\tau \in [0,\tau_{0}\right)$.

**Scenario 2.** Next, we analyze the behavior of the system when the delay approaches the critical value. When $\tau \approx \tau_{0}$, Figure 3d,e show that the system trajectories no longer contract rapidly near $E_{1}$, but gradually exhibit nearly closed oscillatory orbits. In other words, although the trajectories have not yet formed an obvious large-amplitude stable limit cycle, their convergence rate has already been significantly reduced, and critical oscillatory features appear near the equilibrium. This phenomenon indicates that when τ approaches $\tau_{0}$, the system reaches the stability boundary, and the attraction of $E_{1}$ begins to weaken. In other words, what Figure 3d,e depict is neither a completely stable equilibrium state nor a fully developed limit cycle, but rather the critical stage at which the system transitions from a stable equilibrium to periodic oscillation. This phenomenon fully verifies the theoretical conclusion in Theorem 2 that the system undergoes a Hopf bifurcation at $E_{1}$ when τ increases to the critical value $\tau_{0}$.

**Scenario 3.** Finally, we consider the case where the delay exceeds the critical value. We set $\tau =\left(1+\delta \right)\tau_{0}>\tau_{0}$, where δ>0 is a small positive constant. Figure 3f shows that the state variables of the system no longer converge to constant values, but instead exhibit sustained oscillations around the positive equilibrium. Among them, the periodic fluctuations of $L_{1}\left(t\right)$ and $L_{2}\left(t\right)$ are the most pronounced, while C(t), H(t), R(t), and S(t) also undergo small-amplitude oscillations around their respective positive values. This indicates that the system has shifted from the original stable propagation stage to a stage of periodic oscillatory propagation, and the rumor-prevailing equilibrium $E_{1}$ is no longer stable. Figure 3g,h show that the trajectories no longer return to $E_{1}$, but remain in a bounded closed region. Thus, for this fixed-delay parameter setting, bounded oscillatory behavior is observed after $E_{1}$ loses local stability. This numerical observation supports the local Hopf bifurcation analysis, but it should not be interpreted as a proof of the uniqueness or global stability of a limit cycle for all $\tau >\tau_{0}$.

Taken together, Figure 3a–h show that the response delay τ directly affects the local stability of the rumor-prevailing equilibrium $E_{1}$. For $0\leq \tau <\tau_{0}$, the system remains locally stable; when τ crosses $\tau_{0}$, the fixed-delay system may shift from stable propagation to bounded oscillatory propagation. However, this conclusion is derived under the fixed-delay assumption. If the response delay depends on rumor complexity, virality, public attention, or platform amplification, for example τ=τ(q(t)), the model becomes a state-dependent or time-varying delay system, and multiple stability switches, multi-periodic oscillations, quasi-periodic behavior, or chaotic-like fluctuations may occur.

The critical delay $\tau_{0}$ derived above should be interpreted as a mean-field critical delay in an aggregate statistical sense. If a scale-free or multilayer topology is introduced explicitly, high-degree bilingual hubs and inter-layer links may strengthen the bridging effect of the coupled spreader state *C*, so $R_{0}$, the Hopf bifurcation conditions, and $\tau_{0}$ may depend on network quantities such as the degree distribution, the spectral radius of the adjacency matrix, and the coupling strength between language layers. In many parameter regimes, this may reduce the stability margin of $E_{1}$, make $\tau_{0}$ smaller, or even lead to multiple delay-instability thresholds; a rigorous network-structured extension will be considered in future work.

### 4.3. Numerical Verification of the Robustness of the System to Delay Perturbations When $R_{0}<1$

Because Theorem 1 establishes the stability of the rumor-free equilibrium only for the delay-free case τ=0 with $R_{0}\leq 1$, whereas Theorem 2 addresses the delayed system around the rumor-prevailing equilibrium, the case $R_{0}<1$ with τ>0 has not been established analytically in the present framework. We therefore provide numerical evidence to examine whether the rumor-free threshold conclusion remains qualitatively robust under moderate delay perturbations. It should be emphasized that this subsection should be interpreted as a numerical complement rather than a formal extension of Theorem 1.

Before presenting the numerical test, we provide a local analytical explanation. When τ=0 and $R_{0}<1$, Theorem 1 shows that the rumor-free equilibrium $E_{0}$ is asymptotically stable in the delay-free system. Since $R_{0}<1$ is a strict threshold condition, the dominant characteristic roots of the linearized delay-free system are separated from the imaginary axis. For retarded delay differential equations, characteristic roots depend continuously on the delay parameter. Therefore, there exists a sufficiently small $\tau^{*}>0$ such that $E_{0}$ remains locally asymptotically stable for $0\leq \tau <\tau^{*}$. In addition, near $E_{0}$, the delayed terms $\theta_{i}L_{i}\left(t-\tau \right)$, i=1,2, only postpone the transition from monolingual exposed states to the coupled spreader state *C*, and do not create additional rumor-generation sources. This provides a partial analytical reason why a sufficiently small response delay is unlikely to destabilize $E_{0}$ when $R_{0}<1$.

To compare the system responses under delays of different magnitudes in a unified manner, we first construct a reference case with $R_{0}^{ref}>1$ within the same parameter family and compute its critical delay scale $\tau_{0}$. Except for α and β, the remaining parameters are set as Λ=0.20, μ=0.20, $\eta_{1}=0.30$, $\eta_{2}=0.20$, $\theta_{1}=0.50$, $\theta_{2}=0.40$, σ=0.25, ψ=0.12, γ=0.18, δ=0.18, φ=0.20, and ζ=0.10, for which $R_{0}^{ref}=1.25>1$. By linearizing the rumor-prevailing equilibrium $E_{1}$ and solving the characteristic equation, we obtain the first critical delay at which Hopf bifurcation occurs, namely, $\tau_{0}=6.027$. Subsequently, only the propagation parameters are adjusted to α=β=0.45, so that the main experimental setting satisfies $R_{0}=0.98<1$. It should be emphasized that $\tau_{0}$ here is used only as a unified delay-scale reference for comparing the system responses under small delays, delays of critical magnitude, and delays slightly above the critical magnitude; it does not mean that there exists a bifurcation threshold of the same significance in the case $R_{0}<1$.

**Figure 4:**
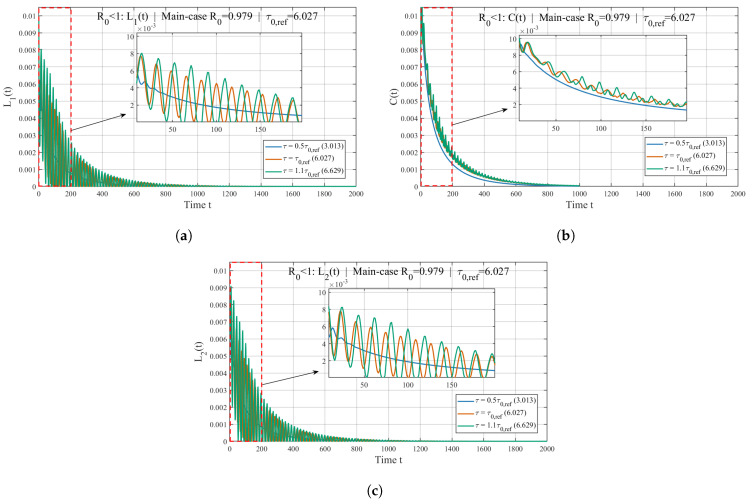
Time-delay robustness analysis under $R_{0}<1$: (**a**) $L_{1}$; (**b**) *C*; (**c**) $L_{2}$.

As shown in Figure 4a–c, under the three delay settings $\tau =0.5\tau_{0}=3.013$, $\tau =\tau_{0}=6.027$, and $\tau =1.1\tau_{0}=6.629$, although the rumor-related states differ in their initial fluctuation amplitudes, oscillation frequencies, and decay rates, their long-term trends are consistent: they all gradually decrease over time and eventually approach zero. In other words, when $R_{0}<1$, even if the delay increases from a relatively small value to a level close to the critical scale, or even slightly exceeds this reference scale, the state variables directly associated with rumor propagation in the system still cannot be maintained at positive levels in the long run. From the perspective of practical propagation, this indicates that in the bilingual coupled rumor propagation system, as long as the basic reproduction number is controlled below 1, rumors still cannot persist in the system over the long term, even when a certain response delay exists in the process of cross-lingual comprehension and re-expression. In other words, although delay may lead to more complex transient fluctuations in the short term, it does not easily reverse the overall trend that rumors eventually die out.

In summary, the numerical results in Figure 4a–c show that when $R_{0}<1$, variations in the delay parameter mainly affect the transient oscillation amplitude, frequency, and decay rate, but the rumor-related states still eventually approach zero under the tested delay values. Combined with the local perturbation argument above, these results suggest that the rumor-free threshold conclusion is qualitatively robust under sufficiently small delays and under the tested moderate delay perturbations. However, this conclusion should not be interpreted as a global stability theorem for all possible delay values.

### 4.4. Parameter Sensitivity Analysis Based on $R_{0}$

The stability analysis in Section 3 and the preceding numerical simulations have shown that $R_{0}$ is the key threshold that determines the long-term evolution direction of the system. To further reveal how each parameter affects the rumor propagation threshold, in this subsection we take the basic reproduction number $R_{0}$ as the indicator and conduct a systematic parameter sensitivity analysis. The aim is to investigate how the parameters of the system govern the variation of $R_{0}$, and how their joint effects drive the system across the threshold boundary, thereby further transforming the theoretical threshold conclusion into interpretable and quantifiable conclusions on parameter regulation.

**Figure 5:**
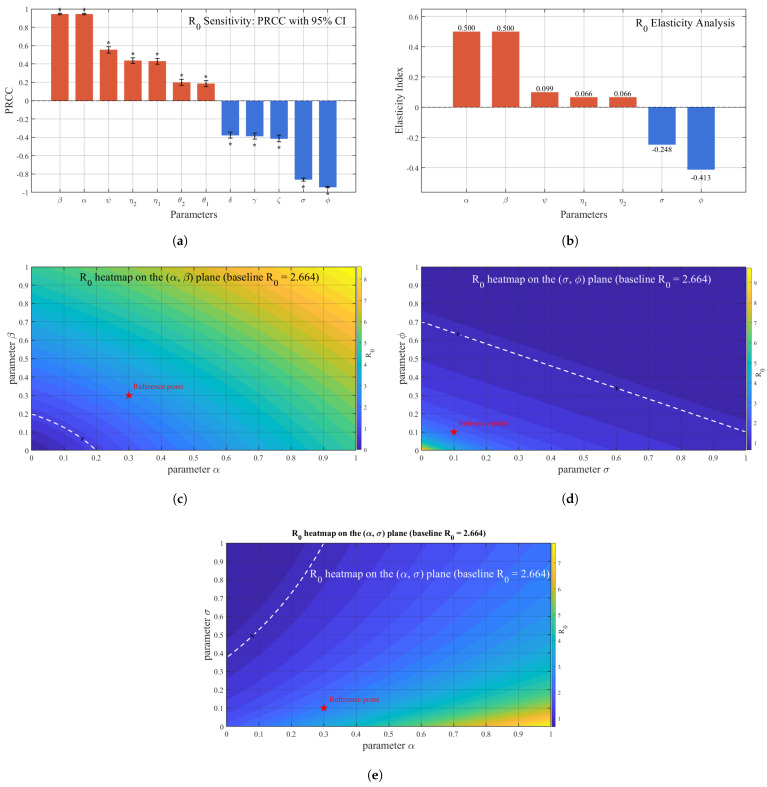
Parameter sensitivity analysis based on $R_{0}$: (**a**) $R_{0}$ Sensitivity: PRCC with 95% CI, asterisks indicate statistically significant PRCC values at the p<0.01 level; (**b**) $R_{0}$ Elasticity analysis, red and blue bars denote positive and negative effects, respectively; (**c**) $R_{0}$ heatmap on the $\left(\alpha ,\beta \right)$ plane, the dashed curves denote the threshold contour $R_{0}=1$; (**d**) $R_{0}$ heatmap on the $\left(\sigma ,\varphi \right)$ plane; (**e**) $R_{0}$ heatmap on the $\left(\alpha ,\sigma \right)$ plane.

This subsection proceeds from three levels of analysis. First, Figure 5a presents the parameter sensitivity results based on the partial rank correlation coefficient (PRCC) and the 95% confidence interval, in order to identify and screen the key parameters that significantly affect $R_{0}$ as a whole, and to determine the direction and relative strength of their effects. Second, for the preliminarily screened key parameters, Figure 5b further adopts the elasticity analysis method to quantify their local relative sensitivities, thereby characterizing the marginal effect intensity of small variations in these parameters on $R_{0}$. It should be emphasized that the elasticity indices in Figure 5b are local sensitivity measures evaluated at the baseline parameter set. They describe the relative change in $R_{0}$ caused by an infinitesimal relative perturbation of one parameter while the other parameters are fixed. Therefore, elasticity analysis complements, but does not replace, PRCC analysis: PRCC provides a global rank-based sensitivity measure over sampled parameter ranges, whereas elasticity describes local marginal effects near a specified baseline. In our results, the two methods are consistent in the main qualitative conclusions: α and β increase $R_{0}$, while σ and φ decrease $R_{0}$. The exact elasticity magnitudes may vary with the baseline; a robustness check under another $R_{0}>1$ baseline is reported in Appendix C, Table A3. Finally, Figure 5c–e present the heatmaps of $R_{0}$ on the three parameter planes (α,β), (σ,φ), and (α,σ), respectively. Through the color distribution, the heatmaps display the overall variation trend of $R_{0}$, and use the contour line $R_{0}=1$ to divide the parameter plane into a spreading region and a non-spreading region, thereby revealing the synergistic enhancement relationship among key parameters, the joint suppression relationship, and the antagonistic relationship between propagation factors and control factors. It should be noted that the same set of baseline parameters is adopted throughout this subsection, namely, Λ=μ=0.05, α=β=0.30, $\eta_{1}=\eta_{2}=0.80$, $\theta_{1}=\theta_{2}=0.55$, γ=δ=0.08, and σ=ψ=φ=ζ=0.10, for which the baseline value is approximately $R_{0}=2.66>1$. On this basis, only the target parameters under consideration are varied, while all the other parameters are kept unchanged, so as to ensure consistency in the analytical framework across different figures.

To make the PRCC analysis reproducible, we used Latin hypercube sampling to generate N=4000 parameter sets. Each sampled parameter was independently drawn from a uniform distribution over $\left[0.8p_{0},1.2p_{0}\right]$, where $p_{0}$ is the baseline value specified above. The parameters Λ and μ were fixed, while the behavioral and coupling parameters $\alpha ,\beta ,\eta_{1},\eta_{2},\theta_{1},\theta_{2},\sigma ,\psi ,\gamma ,\delta$, φ,ζ were sampled. All sampled parameter sets were checked to ensure positive transition rates and $k_{C}>0$. The 95% confidence intervals of PRCC values were obtained from 1000 bootstrap resamples. Rank scatter patterns were also inspected, and no strong non-monotonic relationship was observed for the dominant parameters within the sampled ranges.

As shown in Figure 5a, the PRCC values of parameters α and β are the largest, and both are significantly positive, indicating that they are the most critical factors driving the increase in $R_{0}$. The parameters ψ, $\eta_{1}$, and $\eta_{2}$ also exhibit clear positive effects, but their strengths are weaker than those of α and β. In contrast, the PRCC values of φ and σ are significantly negative and have relatively large absolute magnitudes, indicating that they play a pronounced role in reducing $R_{0}$. The elasticity analysis in Figure 5b further shows that the elasticity coefficients of α and β are both 0.50. Their local effects on $R_{0}$ are therefore almost identical, making them the most sensitive positive parameters in the system. The elasticity coefficients of φ and σ are approximately −0.413 and −0.248, respectively, indicating that strengthening these two inhibitory mechanisms can effectively suppress the propagation threshold. By comparison, although ψ, $\eta_{1}$, and $\eta_{2}$ also increase $R_{0}$, their effects are clearly weaker.

Figure 5a, the heatmap on the (α,β) plane, shows that as α and β increase simultaneously, $R_{0}$ exhibits a pronounced overall upward trend across the parameter plane, and the threshold boundary $R_{0}=1$ is located in the lower-left region. This means that once the two propagation pathways are strengthened simultaneously, the system can easily cross the propagation threshold and enter a high-risk region. Figure 5d, the heatmap on the (σ,φ) plane, shows that as σ and φ increase synchronously, $R_{0}$ decreases significantly, and the region with $R_{0}<1$ expands accordingly, indicating that the two inhibitory mechanisms have a strong joint control effect. Figure 5e, the heatmap on the (α,σ) plane, further reveals the antagonistic relationship between propagation factors and control factors: when α is large, if σ remains at a low level, the system easily stays in the region $R_{0}>1$; however, when σ increases to a sufficiently high level, the system may return to the region $R_{0}<1$ even if α remains large.

It can thus be seen that α and β are the core driving factors that promote rumor propagation, whereas φ and σ are the most important inhibitory factors. From a practical perspective, this means that rumor governance in multilingual environments should not focus only on a single propagation pathway, but should simultaneously suppress the diffusion intensity across different language channels [41,42]. Meanwhile, mechanisms such as information purification, rumor correction, and propagation blocking should also be strengthened so as to enhance the negative regulation within the system. In other words, only by making coordinated efforts in both “reducing propagation parameters” and “strengthening inhibitory parameters” can the system be controlled more effectively within the safe range $R_{0}<1$. It is particularly worth noting that both φ and σ are directly associated with the cross-lingual coupled spreaders *C*. Compared with the monolingual propagation-related states $L_{1}$ and $L_{2}$, state *C* has stronger bridging and diffusion capabilities: it not only connects different language groups, but also more easily amplifies the spread range of rumors in cross-lingual environments. Therefore, from the perspective of control efficiency, effectively suppressing cross-lingual coupled spreaders *C* can often weaken the overall propagation capability of the system more rapidly than merely suppressing $L_{1}$ or $L_{2}$. These results not only verify the dominant role of key parameters in determining the threshold, but also further indicate that prioritizing the control of cross-lingual coupled spreaders is one of the most efficient priority intervention strategies.

### 4.5. Analysis of Rumor-Spreading Effects and Damage Based on Social-Impact Indicators

At present, we have discussed the dynamical properties of the system from the perspectives of the delay-free case, delay-induced bifurcation, and $R_{0}$-based sensitivity analysis, and have clearly identified α, β, σ, and φ as the key parameters affecting the propagation behavior of the system. In connection with the actual process of rumor propagation, threshold analysis of the rumor propagation system is an important means of determining whether rumors persist [43]. However, under the condition $R_{0}>1$, where rumors cannot die out naturally, the assessment of the social-impact and damage caused by rumor propagation is often of greater concern. Therefore, on the basis of the preceding analysis, this subsection further evaluates the model from the perspective of social impact, systematically examining the effects of the above four key parameters on rumors and then analyzing the possible social consequences caused by rumor propagation. Specifically, we further introduce social impact indicators such as the peak spread size, time to peak, and cumulative damage to characterize the combined effects of key parameters on rumor-spreading intensity, propagation rhythm, and the overall degree of harm.

**Figure 6:**
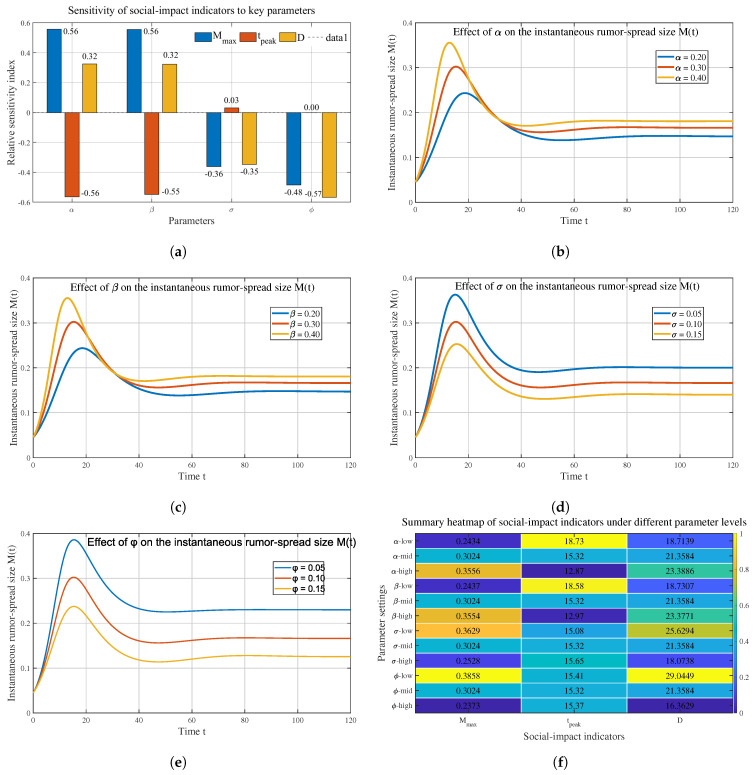
Social-impact assessment of key parameters on rumor spreading: (**a**) sensitivity of social-impact indicators to key parameters; (**b**) effect of α on the instantaneous rumor-spread size M(t); (**c**) effect of β on the instantaneous rumor-spread size M(t); (**d**) effect of σ on the instantaneous rumor-spread size M(t); (**e**) effect of φ on the instantaneous rumor-spread size M(t); (**f**) Summary heatmap of social-impact indicators under different parameter levels.

To this end, we define the instantaneous rumor-spread size as $M\left(t\right)=L_{1}\left(t\right)+L_{2}\left(t\right)+C\left(t\right)$, where $L_{1}$ and $L_{2}$ represent the propagation-related states in the two language transmission pathways, respectively, and *C* represents cross-lingual coupled spreaders. Thus, M(t) is used to characterize the active spreading intensity of the system at time *t*. The equal-weight definition of M(t) is adopted as a conservative aggregate measure of active rumor-spreading intensity, because the present model does not contain empirical information for calibrating the relative amplification weight of coupled spreaders. Since $L_{1}\left(t\right)$, $L_{2}\left(t\right)$, and C(t) remain separately available as model states, M(t) should be interpreted as a baseline aggregate indicator rather than as a claim that all spreader types have identical social influence. If platform data indicate that coupled spreaders have stronger cross-lingual influence, a weighted indicator can be introduced as $M_{\omega }\left(t\right)=L_{1}\left(t\right)+L_{2}\left(t\right)+\omega C\left(t\right)$, ω>1. Under this weighted definition, the contribution of C(t) would be amplified, and the inhibitory roles of *C*-related parameters such as σ and φ in reducing social impact would become more pronounced. Based on M(t), we further construct three social-impact indicators: the peak spread size $M_{\max}=\max_{t}M\left(t\right)$, which is used to measure the maximum impact intensity during rumor propagation; the time to peak $t_{peak}=\underset{t}{\arg\max}M\left(t\right)$, which is used to characterize the time required for the rumor to reach its strongest spreading state, thereby reflecting the length of the governance window; and the cumulative damage index $D=\int_{0}^{T}M(t)dt$, which is used to comprehensively measure the social burden jointly caused by spreading intensity and propagation duration. In the social-impact simulations in Figure 6, the integration horizon is set to T=120 model time units. This horizon covers the initial growth, peak formation, and post-peak stabilization or decline of M(t) under the baseline parameter setting, and therefore represents the main active lifetime of a model rumor episode. Thus, *D* should be interpreted as the cumulative damage accumulated over this fixed observation window. Figure 6a presents a comparative local sensitivity analysis of the four key parameters α, β, σ, and φ with respect to these three social indicators. Figure 6b–e show, respectively, the effects of α, β, σ, and φ on the time evolution of M(t) under different values. Figure 6f, in the form of a summary heatmap, provides a centralized quantitative comparison of $M_{\max}$, $t_{peak}$, and *D* corresponding to these four parameters at the three levels of low, mid, and high. To ensure consistency in the analytical framework across all figures in this subsection, we adopt the same set of baseline parameters throughout, namely, Λ=μ=0.05, α=β=0.30, $\eta_{1}=\eta_{2}=0.80$, $\theta_{1}=\theta_{2}=0.55$, γ=δ=0.08, and σ=ψ=φ=ζ=0.10. On this basis, when only the target parameter under investigation is adjusted individually, all other parameters are kept unchanged.

As shown in Figure 6a, α and β exhibit the most significant positive effects on all three social-impact indicators. Their relative sensitivities to the peak spread size $M_{max}$ are both approximately 0.56, their relative sensitivities to the time to peak $t_{\mathrm{peak}}$ are significantly negative, approximately −0.56 and −0.55, respectively, and their sensitivities to the cumulative damage *D* are also around 0.32. This indicates that as α and β increase, rumor propagation not only reaches its peak more rapidly, but also produces a higher spreading peak, thereby causing more severe overall damage. The time-series results in Figure 6b,c further verify this point: as α or β increases from a low level to a high level, the curve of M(t) shifts upward as a whole, the peak rises markedly, and the time required to reach the peak is significantly shortened, indicating that the strengthening of either language transmission pathway accelerates rumor diffusion and amplifies social impact.

By contrast, Figure 6a shows that σ and φ play significant negative regulatory roles. Among them, φ has the most pronounced inhibitory effect on the cumulative damage *D*, with a sensitivity of approximately −0.57, and it also exerts a relatively strong negative effect on the peak spread size $M_{max}$. The negative effect of σ is secondary, but it can likewise significantly reduce the spreading peak and mitigate the overall damage. Figure 6d,e further show that when σ or φ increases, the peak of M(t) declines markedly, the entire curve shifts downward, and the cumulative damage is correspondingly alleviated. In particular, φ has the most significant suppressive effect on *D*, whereas σ is reflected more in its role of reducing the peak and mitigating the damage. Figure 6f further summarizes these results: α-high and β-high correspond to higher $M_{max}$ and *D*, together with shorter $t_{\mathrm{peak}}$, indicating that the enhancement of propagation parameters significantly increases outbreak intensity and compresses the governance window; by contrast, σ-high and φ-high correspond to lower peaks and smaller cumulative damage, indicating that strengthening the control parameters can effectively alleviate the spreading impact. It is particularly noteworthy that the *D* corresponding to φ-low is the highest in the entire figure, whereas the *D* corresponding to φ-high is the lowest, which fully demonstrates that φ has a very strong regulatory effect in suppressing the overall social damage. To examine whether this conclusion depends on the selected integration horizon, we also recomputed *D* for T=80 and T=160; the qualitative ranking remains unchanged, as shown in Appendix D, Table A4.

From the perspective of the actual rumor propagation process, the two language transmission pathways represented by α and β are the main driving factors that amplify social impact. The larger their values are, the more easily rumors form a peak within a short period of time, thereby compressing the response time available to platforms and society. By contrast, the control mechanisms corresponding to σ and φ, especially the regulatory process acting directly on the cross-lingual coupled spreader state *C*, determine whether the system can effectively reduce the peak and mitigate the damage. Since state *C* plays the role of cross-lingual bridging and diffusion amplification, regulating the parameters associated with *C* is, in fact, of greater global significance than merely controlling monolingual propagation pathways. In other words, real-world rumor governance should not stop at reducing the propagation probability along a single pathway, but should give priority to monitoring and weakening those key spreading nodes that possess cross-lingual diffusion capability. This can be achieved by strengthening measures such as information purification, rapid rumor refutation, and cross-platform coordinated intervention, thereby compressing the active duration and influence range of cross-lingual coupled spreaders. It can thus be seen that quantifying the social harm caused by rumor propagation in terms of maximum impact intensity, speed of reaching the peak, and propagation duration is not only feasible, but also provides an effective way to characterize the real social impact of rumor propagation more comprehensively. Overall, the results of this subsection show that propagation parameters mainly determine the speed and intensity of rumor outbreaks, whereas the control parameters associated with the coupled spreader state mainly determine whether the rumor impact can be effectively weakened and whether the overall social damage can be significantly reduced. This not only extends the previous theoretical discussion on threshold and stability, but also provides a quantitative basis for formulating more refined and targeted multilingual rumor prevention and control strategies from the perspectives of social-damage assessment and governance-priority identification. Operationally, the coupled spreader state *C* can be approximated by users who post, repost, translate, or paraphrase semantically similar rumor content across two language streams within a short time window. Such users can be identified in real time by combining language detection, cross-lingual semantic matching, repost or quote-chain tracing, bridge-position features between language communities, and human verification.

Overall, the social-impact analysis shows that increasing α and β raises the peak and cumulative damage of rumor spreading, whereas increasing the *C*-related inhibitory parameters σ and φ reduces the outbreak intensity and cumulative damage. Therefore, the social-impact results are consistent with the $R_{0}$-based sensitivity analysis and suggest that interventions targeting the coupled spreader state *C* are particularly important in cross-lingual rumor governance.

### 4.6. Real-World Aggregate Trend-Fitting Case Study

To examine the empirical plausibility of the proposed delayed S2LCHR model, we conduct a real-world aggregate trend-fitting case study using the multilingual COVID-19-related tweet dataset of Lopez and Gallemore [44]. The purpose of this case study is not to provide a full individual-level calibration, because the available data provide language-level posting volumes rather than direct observations of cross-lingual reposting chains, bilingual re-expression, hesitation, or withdrawal behavior. Instead, it tests whether the S2LCHR model can reproduce the main macroscopic evolution trends of two language streams in a realistic multilingual OSN setting.

We use the monthly average daily tweet volumes of English and Spanish COVID-19-related posts from January 2020 to December 2022, resulting in 36 monthly observations for each language stream. Therefore, the time unit in this subsection is set to one month. Let $y_{en}\left(t_{i}\right)$ and $y_{es}\left(t_{i}\right)$ denote the observed English and Spanish tweet-volume series. The normalization scale is defined as $N_{0}=\max_{i}\left\{y_{\mathrm{en}}\left(t_{i}\right)+y_{\mathrm{es}}\left(t_{i}\right)\right\}$, and the normalized observations are $Y_{en}\left(t_{i}\right)=\frac{y_{en}\left(t_{i}\right)}{N_{0}},Y_{es}\left(t_{i}\right)=\frac{y_{es}\left(t_{i}\right)}{N_{0}}$.

In the dataset used here, $N_{0}=4,800,858$ tweets/day, and the peak of the combined English–Spanish activity occurs at the sixth month.

Since the model states are not directly observable from language-level tweet volumes, we map the observed language streams to the model outputs as $Y_{en}\left(t\right)\approx L_{1}\left(t\right)+C\left(t\right)$ and $Y_{es}\left(t\right)\approx L_{2}\left(t\right)+C\left(t\right)$. Here, $L_{1}\left(t\right)$ and $L_{2}\left(t\right)$ represent propagation-related intensities in the two language streams, while C(t) denotes the latent cross-lingual coupled spreading intensity. The states C(t), H(t), and R(t) are treated as latent states because the dataset does not directly record cross-lingual reposting, hesitation, withdrawal, or forgetting behavior.

To avoid over-parameterization, only α, β, σ, and φ are fitted from the aggregate time series, while the response delay τ is selected by grid search. The remaining parameters are fixed as Λ=μ=0.001, $\eta_{1}=\eta_{2}=1$, $\theta_{1}=\theta_{2}=0.02$, γ=0.018, δ=0.13, and ψ=ζ=0.05. The best fitting score is obtained at τ=0.50 months, with α=0.6457, β=0.6272, σ=0.0050, φ=0.0906, and the corresponding basic reproduction number is $R_{0}=20.28$. The fitting performance is $R^{2}=0.7437$ and NRMSE=0.1495 for the English stream, $R^{2}=0.7406$ and NRMSE=0.1449 for the Spanish stream, and the combined trend-fitting coefficient of determination is $R^{2}=0.8868$.

**Figure 7:**
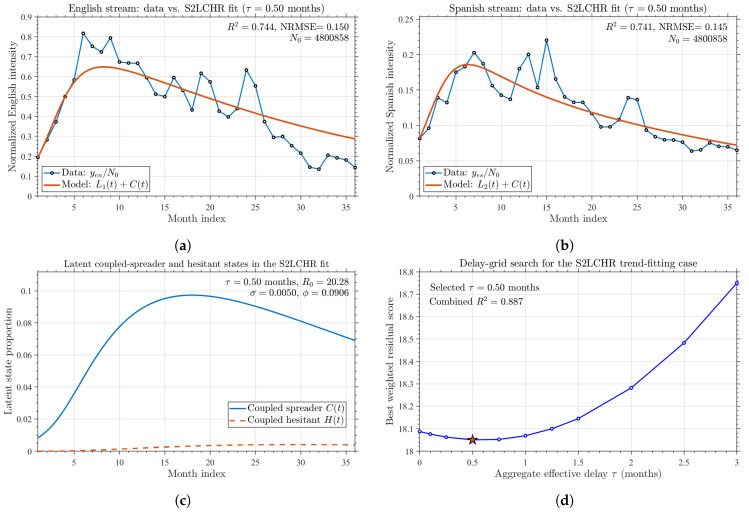
Real-world aggregate trend-fitting case study using multilingual COVID-19-related tweet data: (**a**) English stream, normalized data $y_{en}/N_{0}$ and model output $L_{1}\left(t\right)+C\left(t\right)$; (**b**) Spanish stream, normalized data $y_{es}/N_{0}$ and model output $L_{2}\left(t\right)+C\left(t\right)$; (**c**) fitted latent coupled spreader state C(t) and coupled hesitant state H(t); (**d**) delay-grid search score for selecting the aggregate effective delay τ. The red star marks the selected aggregate effective delay τ=0.50 months obtained from the grid search.

As shown in Figure 7, the fitted curves reproduce the main low-frequency patterns of the two language streams, including the early increase and long-term decline. The latent trajectory of C(t) first increases and then slowly decreases, suggesting an aggregate cross-lingual coupled spreading process behind the observed bilingual tweet streams. The small fitted value σ=0.0050 indicates that the C→H transition is weakly identifiable from monthly language-level posting volumes alone. Since the present dataset provides only aggregate language-level time series, we do not claim full structural identifiability of all parameters; instead, only a subset of aggregate parameters is practically identifiable under fixed baseline assumptions. Therefore, this case study supports the empirical plausibility of the delayed S2LCHR model at the aggregate trend level, but full structural calibration would require richer user-level and event-level data, including user language labels, cross-lingual reposting paths, semantic matching records, intervention timestamps, and behavioral state-transition annotations.

## 5. Conclusions

This paper investigated the coupled propagation of rumors with delay effects in bilingual OSNs. First, it proposed a bilingual coupled rumor propagation framework and, on this basis, incorporated the response delay caused by cross-lingual comprehension, judgment, and re-expression into the model, thereby constructing the time-delay S2LCHR model. Second, it derived the basic reproduction number $R_{0}$ of the system, analyzed the stability of the equilibria, and revealed the dynamical mechanism of delay-induced Hopf bifurcation. Finally, combined with the theoretical results, we carried out extensive numerical simulations, which verified the robustness of the rumor-free equilibrium when $R_{0}<1$, the threshold conclusion, and the delay effects. Meanwhile, based on the sensitivity analysis of $R_{0}$ and the analysis of social-impact indicators, we further characterized the effects of key parameters on propagation trends, spreading intensity, and social damage.

Moreover, the parameter sensitivity analysis showed that the fundamental propagation parameters α and β along the two language transmission pathways are the key promoting factors dominating propagation expansion, whereas the inhibitory parameters σ and φ associated with the coupled spreader state *C* are the most important inhibitory factors. This suggests that the coupled spreader state *C* serves as a critical leverage point for intervention, because it is directly regulated by σ and φ and is indirectly amplified by the upstream transmission parameters α and β. Prioritizing intervention on coupled spreaders can weaken the overall propagation capability of the system more rapidly, thereby reducing propagation risk and social damage. Overall, this paper incorporated the response delay arising in the process of cross-lingual comprehension and re-expression into a unified dynamical framework, and systematically revealed the key role of delay in the propagation threshold, stability transition, and periodic oscillation. At the same time, the conclusion regarding the dominant role of the coupled spreader state provides a quantitative basis and decision support for identifying priority intervention targets and optimizing the allocation of intervention intensity in public risk prevention and control. More broadly, these findings contribute to a system-level understanding of information diffusion and control in complex multilingual online social networks.

Future work will combine the S2LCHR framework with data-driven methods for empirical calibration and adaptive control. Bayesian calibration can estimate key parameters from multilingual rumor time series and cross-lingual reposting records, surrogate models can accelerate DDE-based scenario evaluation, and reinforcement-learning or optimal-control methods can use the S2LCHR system as a mechanistic environment to design adaptive intervention policies.

## Figures and Tables

**Table 2:** Derived composite parameters and constants used in the analysis.

Symbol	Definition	Interpretation
$A_{1}$	$A_{1}=\theta_{1}+\gamma +\mu$	Total outflow rate from the first-language monolingual exposed state $L_{1}$.
$A_{2}$	$A_{2}=\theta_{2}+\delta +\mu$	Total outflow rate from the second-language monolingual exposed state $L_{2}$.
*B*	B=σ+φ+μ	Total outflow rate from the coupled spreader state *C*.
$D_{H}$	$D_{H}=\psi +\zeta +\mu$	Total outflow rate from the coupled hesitant state *H*.
*a*	$a=\alpha \beta (k_{C}\eta_{1}\eta_{2}+\theta_{1}\eta_{2}+\theta_{2}\eta_{1})$	Quadratic coefficient of ${\left(S^{*}\right)}^{2}$ in the equation determining $S^{*}$.
*b*	$b=-k_{C}\left(A_{1}\beta \eta_{2}+A_{2}\alpha \eta_{1}\right)-\left(A_{2}\alpha \theta_{1}+A_{1}\beta \theta_{2}\right)$	Linear coefficient of $S^{*}$ in the quadratic equation determining $S^{*}$.
*c*	$c=k_{C}A_{1}A_{2}$	Constant term in the quadratic equation determining $S^{*}$.
Δ	$\Delta =BD_{H}-\sigma \psi$	Net dissipation term of the *C*–*H* feedback loop.
$k_{H}$	$k_{H}=\frac{\sigma }{\psi +\zeta +\mu }$	Proportional coefficient between $H^{*}$ and $C^{*}$, namely $H^{*}=k_{H}C^{*}$.
$k_{C}$	$k_{C}=\left(\sigma +\varphi +\mu \right)-\psi k_{H}$	Effective removal coefficient of the coupled spreader state after accounting for the H→C feedback.
$\Phi_{1}$	$\Phi_{1}=\frac{\alpha }{A_{1}}\left(\eta_{1}+\frac{\theta_{1}}{k_{C}}\right)$	Contribution of the first-language propagation channel to $R_{0}$.
$\Phi_{2}$	$\Phi_{2}=\frac{\beta }{A_{2}}\left(\eta_{2}+\frac{\theta_{2}}{k_{C}}\right)$	Contribution of the second-language propagation channel to $R_{0}$.
$\Delta_{i}$	i=1,…,6	Routh–Hurwitz determinants of the sixth-degree characteristic polynomial at $E_{1}$ when τ=0.

## Data Availability

The original data presented in the study are openly available at https://zenodo.org/records/20518728 (accessed on 25 May 2026).

## Abbreviations

The following abbreviations are used in this manuscript:

DK	Daley–Kendall
MT	Maki–Thompson
OSNs	Online social networks
PRCC	Partial rank correlation coefficient
S2LCHR	*S*–$L_{1}$–$L_{2}$–*C*–*H*–*R* rumor-spreading model

## Appendix A. Derivation and Feasibility of the Rumor-Prevailing Equilibrium

At equilibrium, the delay terms satisfy $L_{i}\left(t-\tau \right)=L_{i}\left(t\right)$, i=1,2. Using the composite quantities defined in Table 2, the H-equation gives $H^{*}=k_{H}C^{*}$. Substituting this into the *C*-equation gives $\theta_{1}L_{1}^{*}+\theta_{2}L_{2}^{*}=k_{C}C^{*}$. From the $L_{1}$- and $L_{2}$-equations, we have $L_{1}^{*}=\frac{\alpha C^{*}S^{*}}{A_{1}-\alpha \eta_{1}S^{*}}$, $L_{2}^{*}=\frac{\beta C^{*}S^{*}}{A_{2}-\beta \eta_{2}S^{*}}$. Substituting these expressions into $\theta_{1}L_{1}^{*}+\theta_{2}L_{2}^{*}=k_{C}C^{*}$ and assuming $C^{*}>0$, we obtain

$$\frac{\alpha \theta_{1}S^{*}}{A_{1}-\alpha \eta_{1}S^{*}}+\frac{\beta \theta_{2}S^{*}}{A_{2}-\beta \eta_{2}S^{*}}=k_{C}$$

After multiplying both sides by

$$\left(A_{1}-\alpha \eta_{1}S^{*}\right)\left(A_{2}-\beta \eta_{2}S^{*}\right),$$

the above equation can be written as

$$a{\left(S^{*}\right)}^{2}+bS^{*}+c=0,$$

where

$$\begin{array}{cc} a & =\alpha \beta (k_{C}\eta_{1}\eta_{2}+\theta_{1}\eta_{2}+\theta_{2}\eta_{1}),\\ b & =-k_{C}\left(A_{1}\beta \eta_{2}+A_{2}\alpha \eta_{1}\right)-\left(A_{2}\alpha \theta_{1}+A_{1}\beta \theta_{2}\right),\\ c & =k_{C}A_{1}A_{2}. \end{array}$$

Thus, when $b^{2}-4ac\geq 0$ and a≠0, the algebraic candidates are

$$S_{\pm }^{*}=\frac{-b\pm \sqrt{b^{2}-4ac}}{2a}.$$

For any candidate value *S*, define

$$\begin{array}{cc} C(S) & =\frac{\Lambda -\mu S}{S\left(\frac{A_{1}\alpha }{A_{1}-\alpha \eta_{1}S}+\frac{A_{2}\beta }{A_{2}-\beta \eta_{2}S}\right)},\\ L_{1}\left(S\right) & =\frac{\alpha C(S)S}{A_{1}-\alpha \eta_{1}S},\;L_{2}\left(S\right)=\frac{\beta C(S)S}{A_{2}-\beta \eta_{2}S},\\ H(S) & =k_{H}C\left(S\right), \end{array}$$

and

$$R\left(S\right)=1-S-L_{1}\left(S\right)-L_{2}\left(S\right)-C\left(S\right)-H\left(S\right).$$

The admissible root is the one that lies in the feasible set

$$S_{ad}=\left\{S|0<S<\min\left(1,\frac{\Lambda }{\mu },\frac{A_{1}}{\alpha \eta_{1}},\frac{A_{2}}{\beta \eta_{2}}\right),\;C\left(S\right)>0,\;H\left(S\right)>0,\;R\left(S\right)>0\right\}.$$

The upper bounds

$$S<\frac{A_{1}}{\alpha \eta_{1}},\;S<\frac{A_{2}}{\beta \eta_{2}}$$

ensure that the denominators in $L_{1}\left(S\right)$ and $L_{2}\left(S\right)$ are positive. The condition $S<\frac{\Lambda }{\mu }$ helps ensure a positive numerator in C(S). Together with the normalization condition, the positivity of all components implies that the equilibrium lies in the feasible region.

Consequently, the admissible rumor-prevailing equilibrium is constructed as $E_{1}=(S^{*}$, $L_{1}^{*}$, $L_{2}^{*}$, $C^{*}$, $H^{*}$, $R^{*})$, where

$$S^{*}\in \left\{S_{+}^{*},S_{-}^{*}\right\}\cap S_{ad},$$

and

$$\begin{array}{cc} C^{*} & =C(S^{*}),\\ L_{1}^{*} & =L_{1}\left(S^{*}\right)=\frac{\alpha C\left(S^{*}\right)S^{*}}{A_{1}-\alpha \eta_{1}S^{*}},\\ L_{2}^{*} & =L_{2}\left(S^{*}\right)=\frac{\beta C\left(S^{*}\right)S^{*}}{A_{2}-\beta \eta_{2}S^{*}},\\ H^{*} & =H\left(S^{*}\right)=k_{H}C\left(S^{*}\right),\\ R^{*} & =R\left(S^{*}\right)=1-S^{*}-L_{1}^{*}-L_{2}^{*}-C^{*}-H^{*}. \end{array}$$

If only one of the two algebraic roots satisfies the above feasibility conditions, that root is selected to construct $E_{1}$. If both roots satisfy the feasibility conditions, both correspond to feasible rumor-prevailing equilibria and should be analyzed separately. If no algebraic root satisfies the feasibility conditions, then no positive rumor-prevailing equilibrium exists in the feasible region for that parameter set.

## Appendix B. Numerical Verification of the Routh–Hurwitz Conditions

**Table A1:** Numerical verification of the Routh–Hurwitz conditions for the main $R_{0}>1$ parameter sets.

Parameter Set	$R_{0}$	$\Delta_{1}$	$\Delta_{2}$	$\Delta_{3}$	$\Delta_{4}$	$\Delta_{5}$	$\Delta_{6}$	Conclusion
Figure 2, $R_{0}>1$ delay-free case	1.631	1.903	2.113	$7.101\times 10^{-1}$	$4.296\times 10^{-2}$	$2.910\times 10^{-4}$	$1.232\times 10^{-7}$	satisfied
Figure 3, Hopf baseline case	5.475	2.863	6.749	6.140	1.099	$2.377\times 10^{-2}$	$2.000\times 10^{-5}$	satisfied
Figure 4, reference $R_{0}^{\mathrm{ref}}>1$ case	1.250	2.950	7.571	8.084	1.857	$4.810\times 10^{-2}$	$6.925\times 10^{-5}$	satisfied
Figure 5 and Figure 6, sensi-tivity/social-impact baseline	2.664	1.863	1.893	$4.669\times 10^{-1}$	$1.303\times 10^{-2}$	$2.942\times 10^{-5}$	$2.578\times 10^{-9}$	satisfied

## Appendix C. PRCC Sampling Settings

To ensure reproducibility of the PRCC-based sensitivity analysis, the sampling design and parameter ranges are summarized in Table A2. Latin hypercube sampling was used, and all sampled parameters were drawn from uniform distributions centered at the baseline values used in Section 4.4.

**Table A2:** Sampling settings for the PRCC-based sensitivity analysis of $R_{0}$.

Parameter	Baseline $p_{0}$	Sampling Distribution	Sampling Range
α, β	0.30	Uniform	[0.24, 0.36]
$\eta_{1}$, $\eta_{2}$	0.80	Uniform	[0.64, 0.96]
$\theta_{1}$, $\theta_{2}$	0.55	Uniform	[0.44, 0.66]
σ, ψ, φ, ζ	0.10	Uniform	[0.08, 0.12]
γ, δ	0.08	Uniform	[0.064, 0.096]

The sample size was N=4000, and 1000 bootstrap resamples were used to obtain the 95% confidence intervals. The parameters Λ=μ=0.05 were fixed in this PRCC experiment. All sampled parameter sets were checked to ensure positive transition rates and $k_{C}>0$. Rank scatter patterns were inspected to assess PRCC interpretability, and no strong non-monotonic pattern was observed for the dominant parameters α,β,σ, and φ within the sampled ranges.

Baseline A is the parameter set used in the sensitivity and social-impact analyses of Section 4.4, with Λ=μ=0.05, α=β=0.30, $\eta_{1}=\eta_{2}=0.80$, $\theta_{1}=\theta_{2}=0.55$, γ=δ=0.08, and σ=ψ=φ=ζ=0.10. Baseline B is the Hopf-bifurcation baseline used in Section 4.2, with Λ=μ=0.05, α=0.95, β=0.90, $\eta_{1}=\eta_{2}=0.80$, $\theta_{1}=0.60$, $\theta_{2}=0.55$, γ=δ=0.08, σ=0.70, ψ=0.40, and φ=ζ=0.10. The negative values in the table are elasticity indices rather than parameter values. They indicate that increasing the corresponding positive parameters decreases $R_{0}$ near the given baseline.

**Table A3:** Robustness check of local elasticity indices under two positive $R_{0}>1$ baseline parameter sets.

Parameter	Baseline A Value	Elasticity at Baseline A	Baseline B Value	Elasticity at Baseline B	Effect Direction
α	0.30	0.500	0.95	0.516	Promoting
β	0.30	0.500	0.90	0.484	Promoting
$\eta_{1}$	0.80	0.066	0.80	0.098	Promoting
$\eta_{2}$	0.80	0.066	0.80	0.094	Promoting
ψ	0.10	0.099	0.40	0.329	Promoting
σ	0.10	−0.248	0.70	−0.453	Inhibitory
φ	0.10	−0.413	0.10	−0.237	Inhibitory

## Appendix D. Robustness Checks for Social-Impact Indicators

To check whether the cumulative damage ranking depends on the integration horizon *T*, we recomputed $D=\int_{0}^{T}M\left(t\right)\;dt$ for T=80, T=120, and T=160 model time units. The results are summarized in Table A4.

**Table A4:** Robustness of cumulative damage *D* under different integration horizons.

Integration Horizon *T*	Largest *D*	Smallest *D*	Qualitative Conclusion
80	φ-low, D=19.8438	φ-high, D=11.3066	consistent
120	φ-low, D=29.0449	φ-high, D=16.3629	consistent
160	φ-low, D=38.2417	φ-high, D=21.3962	consistent

The ranking remains unchanged across the three integration horizons. Therefore, the inhibitory role of φ in reducing cumulative social damage is robust to moderate changes in the observation window.

## References

[1] Wang J., Liao J., Lu J.G., Li J., Liu M. (2025). Dynamic Analysis of the Multi-Lingual S2IR Rumor Propagation Model Under Stochastic Disturbances. Entropy.

[2] Hou H., Wang Y., Zhai Q., Zhou Z., Jing Y. (2026). Prediction and optimal control of a time-varying SAPDR rumor spreading model via PINNs. Nonlinear Dyn..

[3] Chen C., Lu Y., Wei J., Yu J., Liu S. (2025). Dynamic analysis of rumor propagation considering both hibernation and countering mechanism. Int. J. Mod. Phys. C.

[4] Xie Z., Lan M., Xu T., Pan Y., Wu J., Sun Y., Weng W. (2025). Study on the spreading amplification effect of compound disaster rumors involving multiple public safety events. Fundam. Res..

[5] Khanduzi R., Ebrahimzadeh A., Jajarmi A. (2026). Optimal control of a rumor spreading model in online social networks based on a spectral method and pufferfish optimizer. Int. J. Dyn. Control.

[6] Xia Y., Jiang H. (2024). A stochastic rumor spreading model with event-triggered discontinuous feedback control in multilingual online networks. J. Appl. Math. Comput..

[7] Li X., Lu S., Yu Z., Wu S., Yang F. (2025). Hybrid control strategy and optimal control for rumor spreading. Chaos Solitons Fractals.

[8] Luo X., Jiang H., Chen S., Li J. (2023). Stability and optimal control for delayed rumor-spreading model with nonlinear incidence over heterogeneous networks. Chin. Phys. B.

[9] Zhang G., Dong H., Li H., Xu C., Karimi H.R., Xiao M., Cao J. (2025). Dynamics mechanism of time-delay reaction–diffusion rumor-propagation model with saturation control. Adv. Contin. Discret. Model..

[10] Daley D.J., Kendall D.G. (1964). Epidemics and rumours. Nature.

[11] Daley D.J., Kendall D.G. (1965). Stochastic rumours. IMA J. Appl. Math..

[12] Jia C., Deng G., Chen X., Chen K., Wang R., Li T., Xiao Y. (2026). A dynamic model of rumor propagation based on adversarial behavior and evolutionary games. Expert Syst. Appl..

[13] Polin S.A., Hasan M.N., Islam S., Podder C.N. (2025). Behavioral influences on rumor dynamics: A compartmental model with hesitation, forgetting, and self-remembering mechanisms in complex heterogeneous social networks. Chaos Solitons Fractals.

[14] Li Y., Liu Y., Zhang J. (2025). Dynamic Analysis of a Fractional-Order SINPR Rumor Propagation Model with Emotional Mechanisms. Fractal Fract..

[15] Yu P., Zhang X., Ma L. (2025). Factors influencing users’ rumour spreading intention during public health emergencies: Based on protection motivation theory. Int. J. Mob. Commun..

[16] Yu S., Yu Z., Jiang H., Mei X., Li J. (2020). The spread and control of rumors in a multilingual environment. Nonlinear Dyn..

[17] Wang X., Wang X., Yang W. (2025). A rumor propagation model integrated with psychological factors. Int. J. Biomath..

[18] Yu S., Yang L., Liu L., Yu Z., Jiang H. (2026). The dynamics and control of rumor propagation model with conformity psychology and psychological defense in heterogeneous networks. Nonlinear Dyn..

[19] Yan W., Hu Y. (2026). Cognitive Familiarity-Driven Rumor Diffusion: A Dynamic Compartmental Model With Stratified Susceptibility. Adv. Math. Phys..

[20] Ding X., Xing Q., Deng W., Tian Y. (2025). Dynamics analysis of rumor propagation model in dual-layer heterogeneous social networks considering information coupling effect. Knowl.-Based Syst..

[21] Tong X., Jiang H., Qiu J., Xiong X., Li J. (2026). Stochastic dynamics of rumor cross-layer propagation on multi-layer coupled networks incorporating video and text information. Commun. Nonlinear Sci. Numer. Simul..

[22] Du K., Fan R., Wang D., Xie X., Xu X., Lin J. (2025). Competitive information spreading model in two-layer networks considering dual debunking mechanisms and time lag effects. Phys. A Stat. Mech. Its Appl..

[23] Liu Z., Zhang H., Sun J., Wang L. (2025). Defense Strategy for Social Network Fake News Under Non-Real-Time Data Collection and Disjointed Decision Making. IEEE Trans. Netw. Sci. Eng..

[24] Xia Y., Jiang H., Li J., Yu S. (2025). Dynamic analysis of a fractional-order rumor spreading model with double time delay under higher-order interactions. Nonlinear Anal. Model. Control.

[25] Karumathil K., Kundu S., Konar P. (2026). Dynamic analysis of rumor propagation: Media effect, forgetting and remembering mechanisms. Eur. Phys. J. Plus.

[26] Niu Y., Muhammadhaji A. (2025). Dynamics of a fractional-order IDSR rumor propagation model with time delays. Fractal Fract..

[27] Li D., Zhao Y., Deng Y., Wang Y. (2025). Rumor propagation in the framework of evolutionary game analysis. Chaos Interdiscip. J. Nonlinear Sci..

[28] Mou X., Xiao Y., He W., Wang R., Duan S., Li Q. (2025). An information dissemination model based on the rumor and antirumor and cognitive game. IEEE Trans. Comput. Soc. Syst..

[29] Guo H., Yan X., Zhang J. (2025). Modeling and simulation of rumor propagation and optimal control strategy based on social positive reinforcement. Eur. Phys. J. Plus.

[30] He Q., Zhang Z., Bi T., Fang H., Yi X., Yu K. (2025). Adaptive rumor suppression on social networks: A multi-round hybrid approach. ACM Trans. Knowl. Discov. Data.

[31] Gan C., Yang W., Zhu Q., Li M., Jain D.K., Štruc V., Huang D.W. (2025). Hybrid rumor debunking in online social networks: A differential game approach. IEEE Trans. Syst. Man Cybern. Syst..

[32] Wang Y., Li W., Wang C., Liu M., Yang M. (2026). The dynamics and control of false information spreading model classified by node influence difference. J. Stat. Mech. Theory Exp..

[33] Zhu L., Ding N. (2025). Parameter identification of reaction-diffusion information propagation model based on optimal control and Turing pattern theory. IEEE Trans. Comput. Soc. Syst..

[34] Zhu L., Cao B., Shen S. (2026). Research on the parameter identification of network propagation system based on pattern theory and optimization method. Eng. Appl. Artif. Intell..

[35] Zhu Y., Liu Q., Guo C., Fan T., Lü L. (2026). Structure-aware optimal intervention for rumor dynamics on networks: Node-level, time-varying, and resource-constrained. Chaos Solitons Fractals.

[36] He Q., Gao F., Wang X., Zhao Y., Huang M., Ma L., Zhao L. (2025). Efficient Rumor Suppression With Dynamic Blocking Strategy in Social Networks. IEEE Trans. Comput. Soc. Syst..

[37] Zhong X., Zhang J., Wang A., Liu G., Deng F., Wang J. (2025). Rumor suppression in a three-layer network: A reinforcement learning algorithm. IEEE Trans. Netw. Sci. Eng..

[38] Li P.Y., Hu F., Li F.X., Zhao Y.F., Song Y.R. (2026). SEIAR rumor spreading model with antagonistic states in hypernetworks. Appl. Math. Comput..

[39] Li Q., Jiang F., Sun H., Wang R., Jia C., Li T., Xiao Y. (2025). A rumor dissemination control model based on evolutionary game and multiple user states. IEEE Trans. Netw. Sci. Eng..

[40] Zhou Q., Duan X., Yu G. (2025). Research on dynamic modeling and control mechanisms of rumor spread considering high-order interactions and counter-rumor groups. Chaos Solitons Fractals.

[41] He Q., Tang Z., Jiang R., Zhang Z., Fang H., Wang X., Ma L., Yu K. (2026). Uncertainty Rumor Blocking in Social Networks: A Graph Inverse Reinforcement Learning Approach. IEEE Trans. Netw..

[42] Tong X., Jiang H., Xiong X., Qiu J., Yu S., Li J. (2025). Stochastic dynamics of truth-rumor model incorporating age-related control and time-varying stopping rate under dual media interventions on heterogeneous social networks. Commun. Nonlinear Sci. Numer. Simul..

[43] Wang Z., Zhu L. (2025). Pattern dynamics analysis and control strategies of rumor propagation model in complex networks based on real data. Inf. Sci..

[44] Lopez C.E., Gallemore C. (2021). An Augmented Multilingual Twitter Dataset for Studying the COVID-19 Infodemic. Soc. Netw. Anal. Min..

